# Supplementary material for: The Antimicrobial and Antibiofilm Potential of New Water-Soluble Tris-Quaternary Ammonium Compounds
Source: Int J Mol Sci. 2023 Jun 22;24(13):10512. doi: 10.3390/ijms241310512 (PMC10341745; doi:10.3390/ijms241310512)

Supporting Information

The Antimicrobial and Antibiofilm Potential of New Water-Soluble tris-Quaternary Ammonium Compounds.

Pages: 38

Figures: 6

Tables: 1

Content:

^{1}H, ^{13}C NMR spectra and HRMS of spacer 3a-c 2

^{1}H, ^{13}C NMR and HRMS spectra of Novel pyridinium tris-QACs 8

2D NMR spectra of the compound 7g 33

Figures and tables 36

^{1}H, ^{13}C NMR spectra and HRMS of spacer 3a-c

^{1}H NMR spectrum of 1,3,5-tris(2-(2-chloroethoxy)ethyl)-1,3,5-triazinane-2,4,6-trione **(3a)**

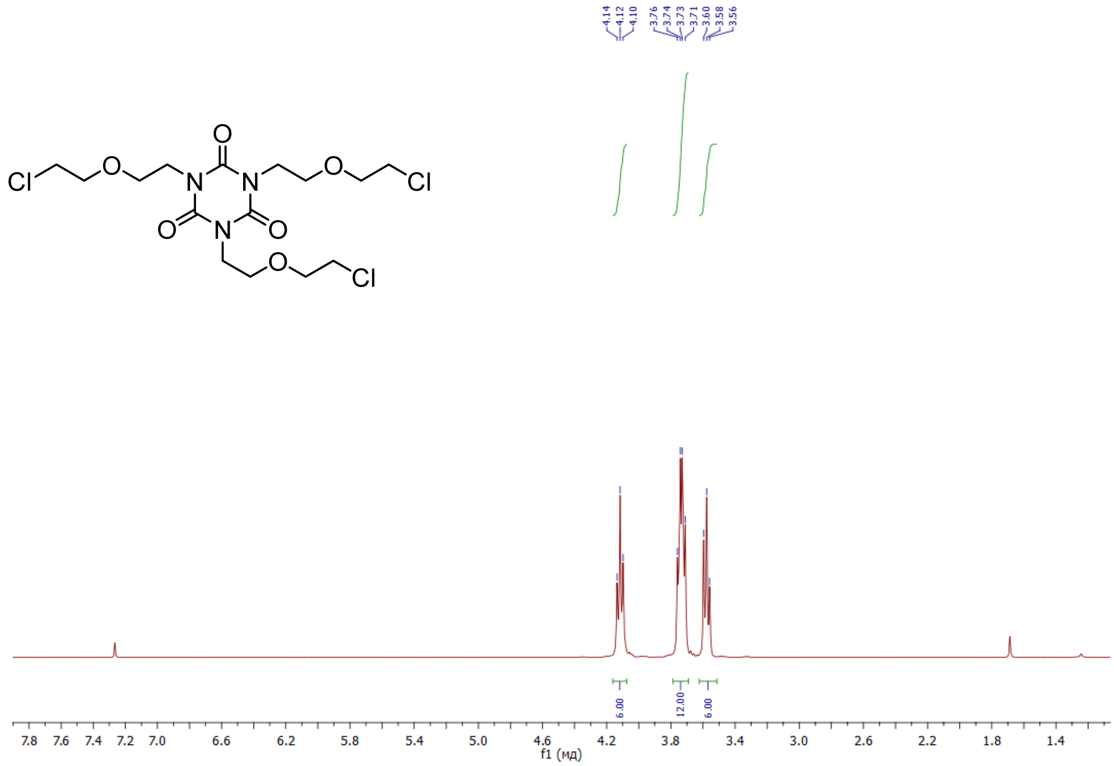

^{13}C NMR spectrum of **3a**

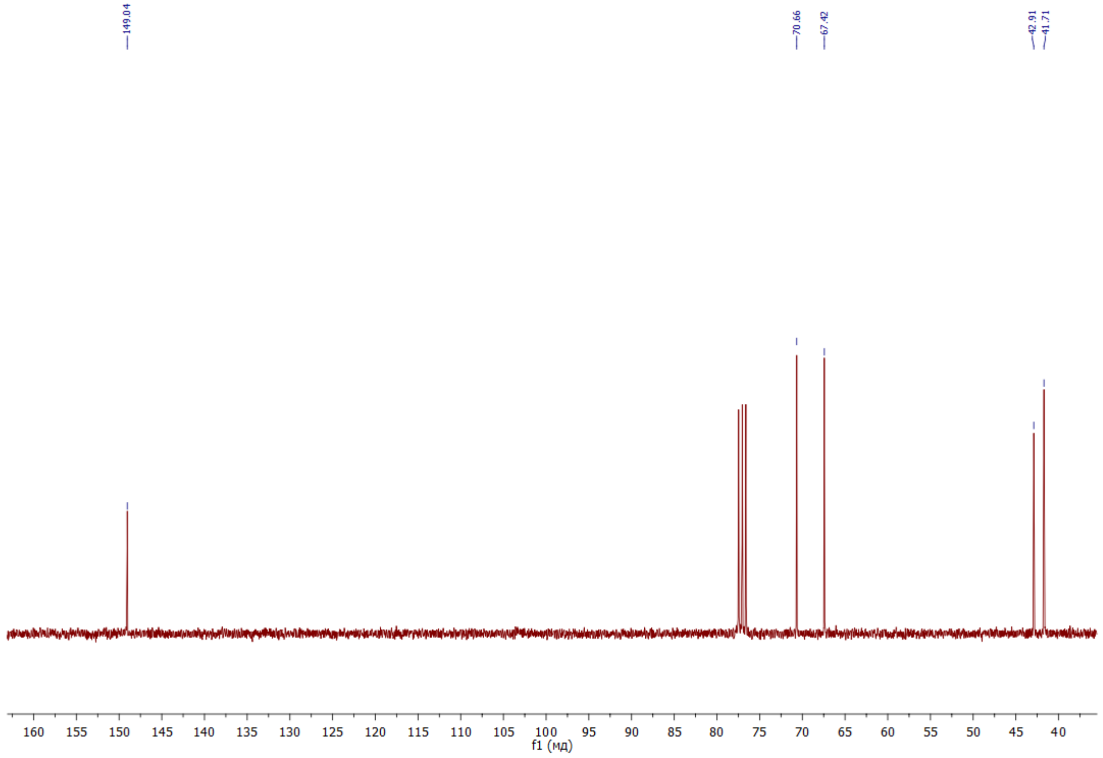

HRMS of **3a**

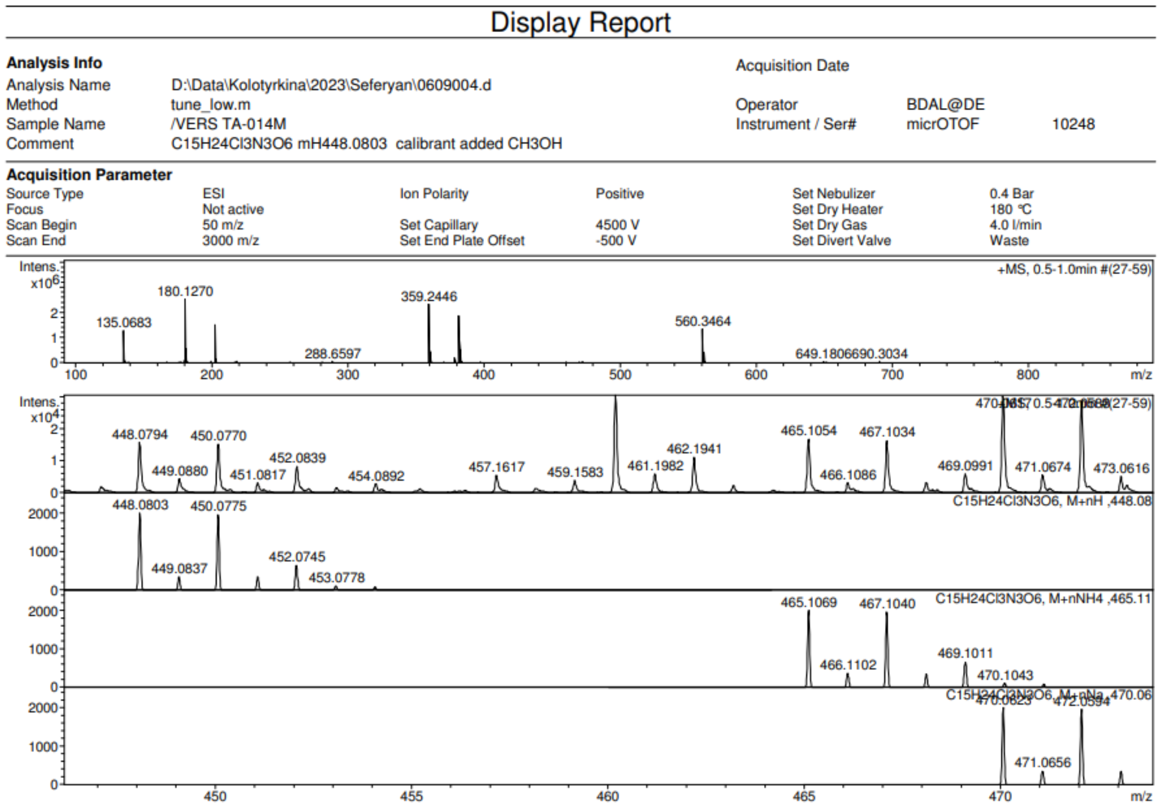

^{1}H NMR spectrum of 1,3,5-tris(2-(2-(2-chloroethoxy)ethoxy)ethyl)-1,3,5-triazinane-2,4,6-trione **(3b)**

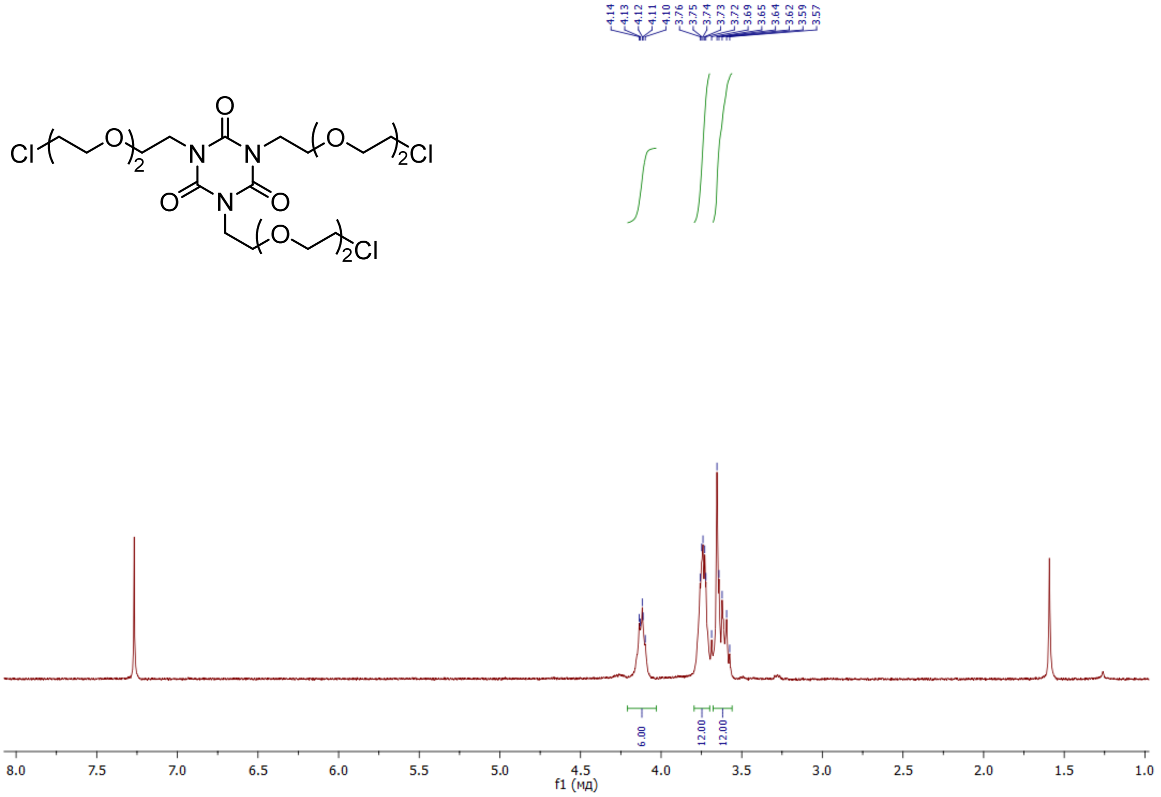

^{13}C NMR spectrum of **3b**

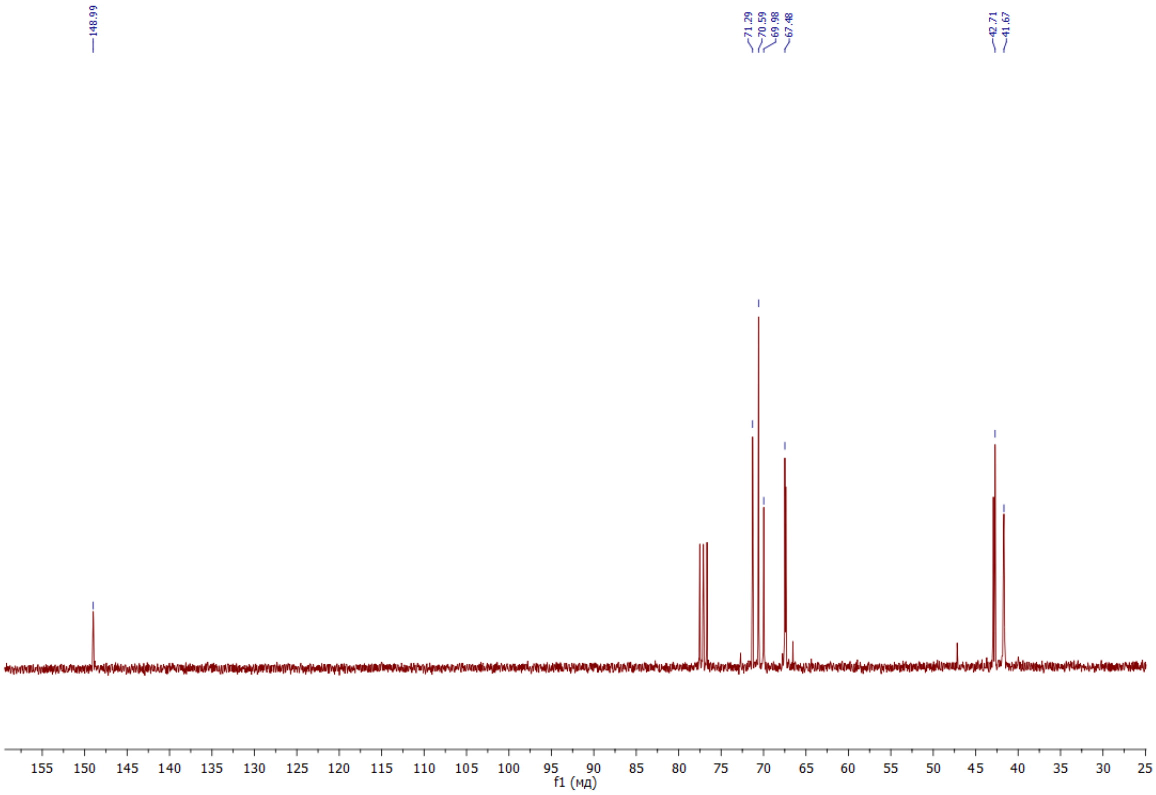

HRMS of **3b**

Display Report

Analysis Info

Analysis Name	D:\Data\Chizhov\Vereshchagin\Seferyan\May_12_2023\ta-025_&clb.d		
Method	tune_wide.m	Operator	BDAL@DE
Sample Name	/VERS TA-025	Instrument / Ser#	micrOTOF 10248
Comment	CH3CN 100 %, dil. 200, calibrant added		

Acquisition Parameter

Source Type	ESI	Ion Polarity	Positive	Set Nebulizer	0.4 Bar
Focus	Not active			Set Dry Heater	180 °C
Scan Begin	50 m/z	Set Capillary	4500 V	Set Dry Gas	4.0 l/min
Scan End	3000 m/z	Set End Plate Offset	-500 V	Set Divert Valve	Waste

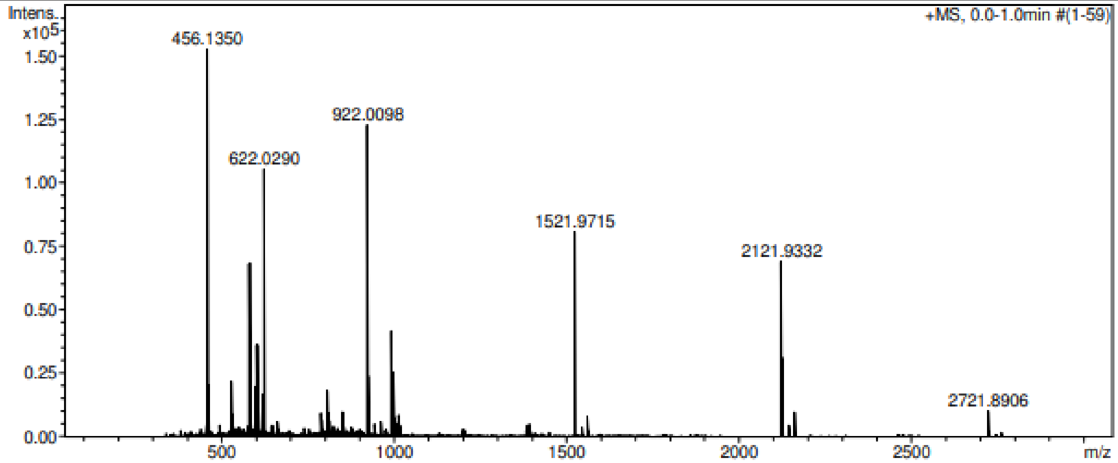

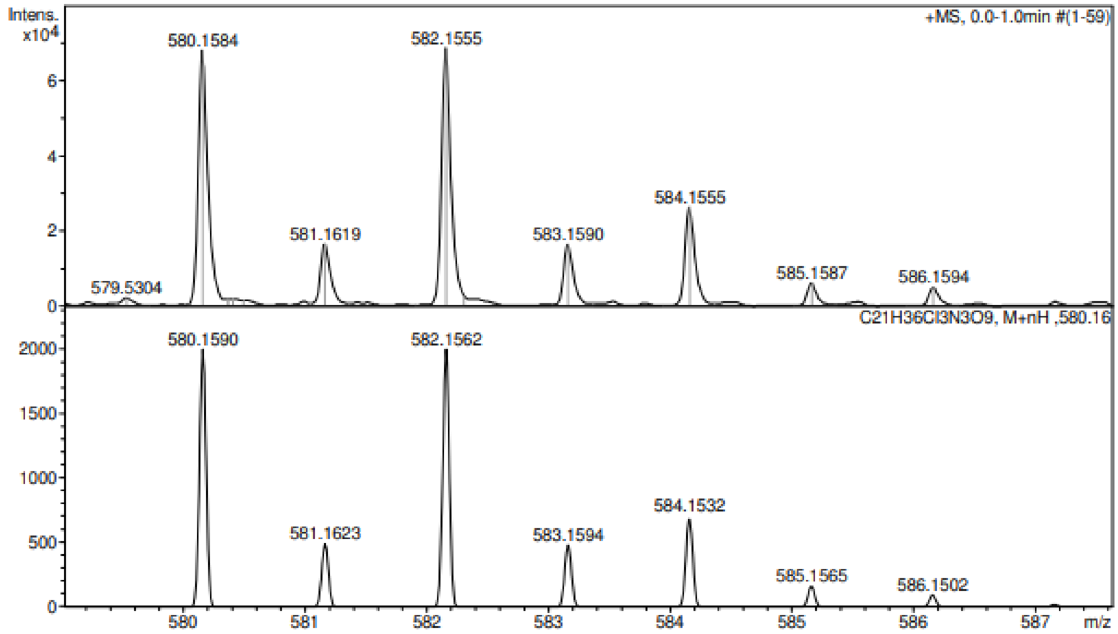

^{1}H NMR spectrum of 1,3,5-tris(2-(2-(2-(2-chloroethoxy)ethoxy)ethoxy)ethyl)-1,3,5-triazinane-2,4,6-trione **(3c)**

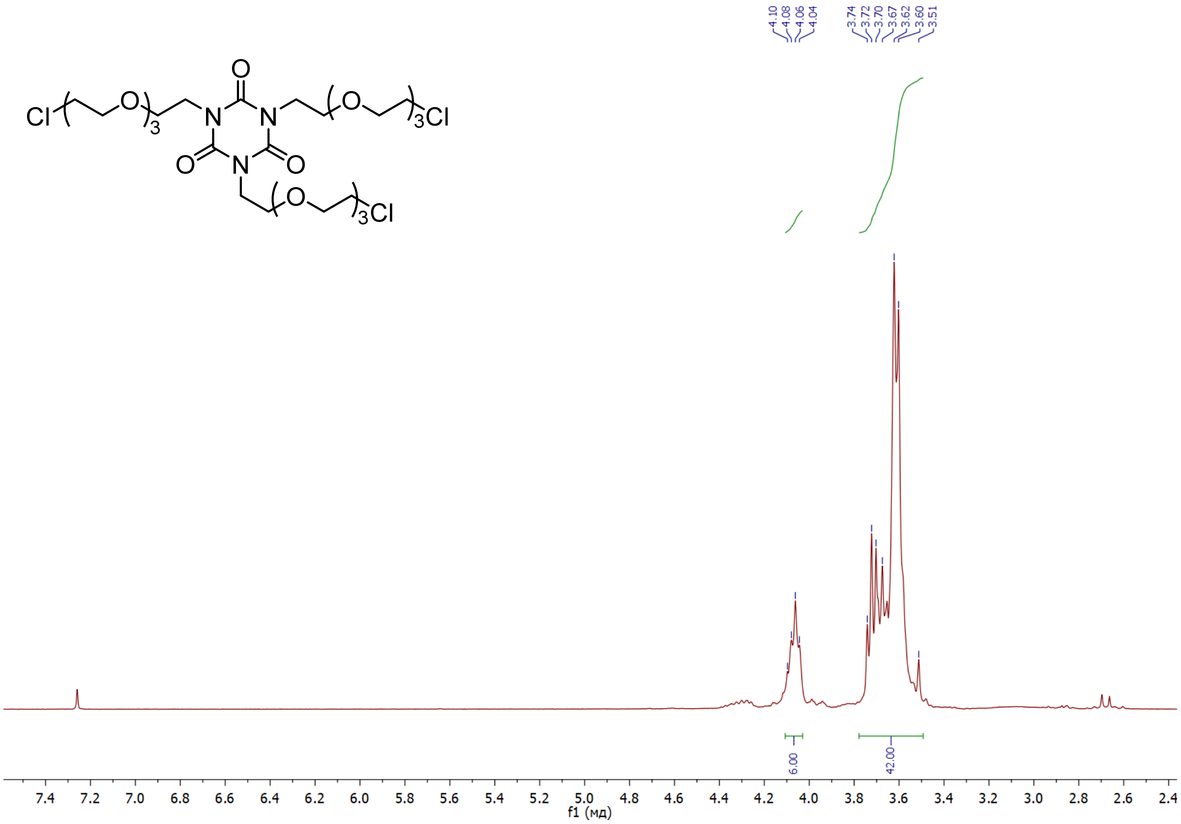

^{13}C NMR spectrum of **3c**

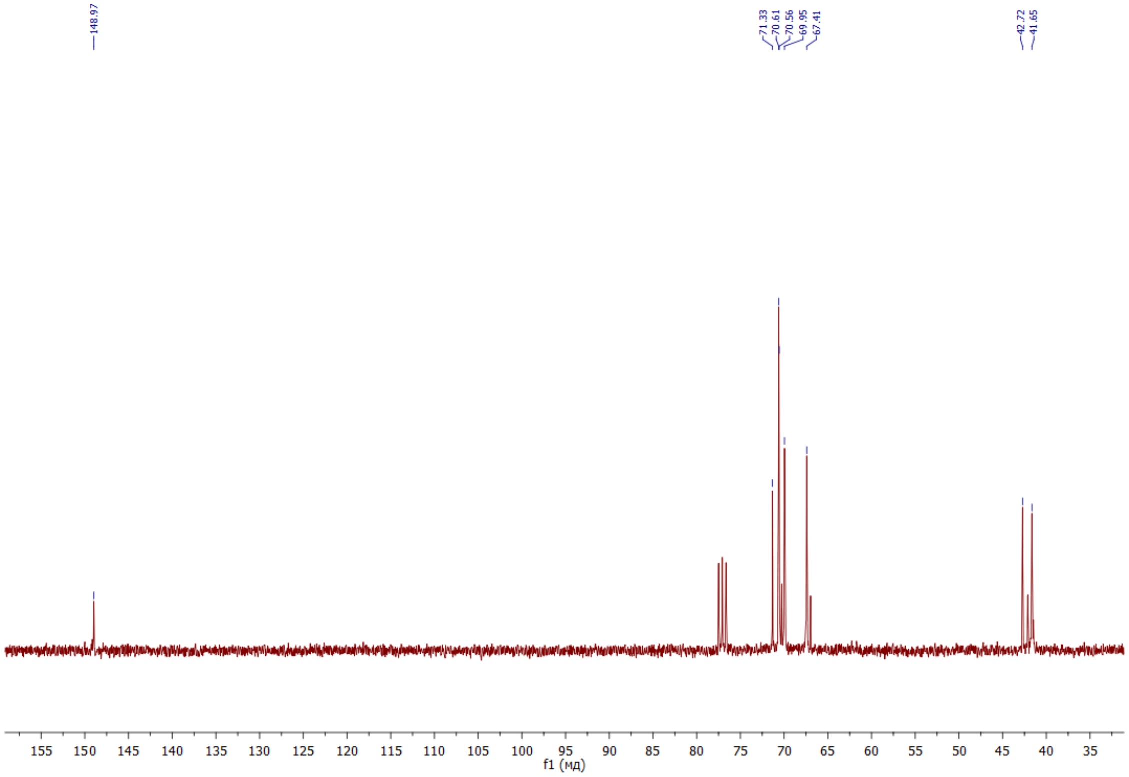

HRMS of 3c

Display Report

Analysis Info

Analysis Name	D:\Data\Chizhov\Vereshchagin\Seferyan\May_12_2023\ta-024_&clb.d		
Method	tune_wide.m	Operator	BDAL@DE
Sample Name	/VERS TA-024	Instrument / Ser#	micrOTOF 10248
Comment	CH3CN 100 %, dil. 200, calibrant added		

Acquisition Parameter

Source Type	ESI	Ion Polarity	Positive	Set Nebulizer	0.4 Bar
Focus	Not active			Set Dry Heater	180 °C
Scan Begin	50 m/z	Set Capillary	4500 V	Set Dry Gas	4.0 l/min
Scan End	3000 m/z	Set End Plate Offset	-500 V	Set Divert Valve	Waste

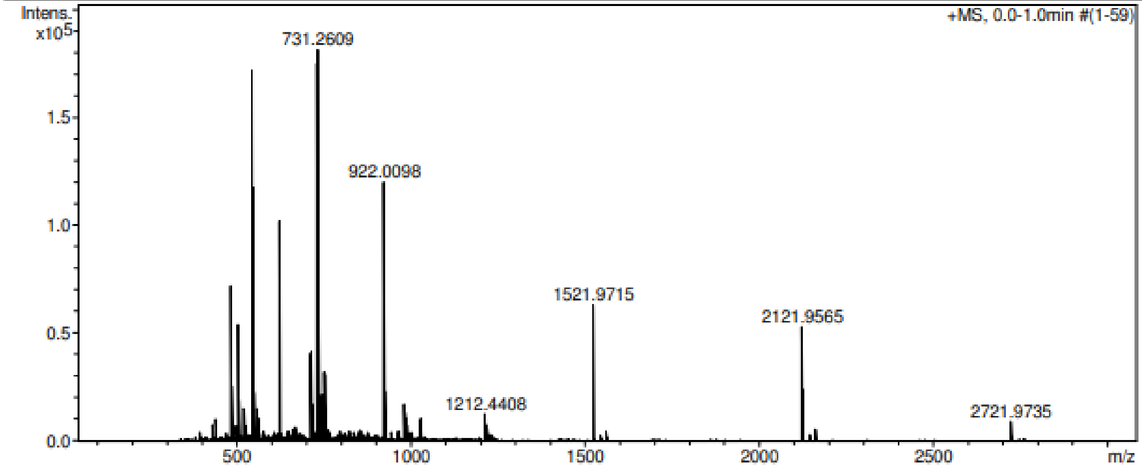

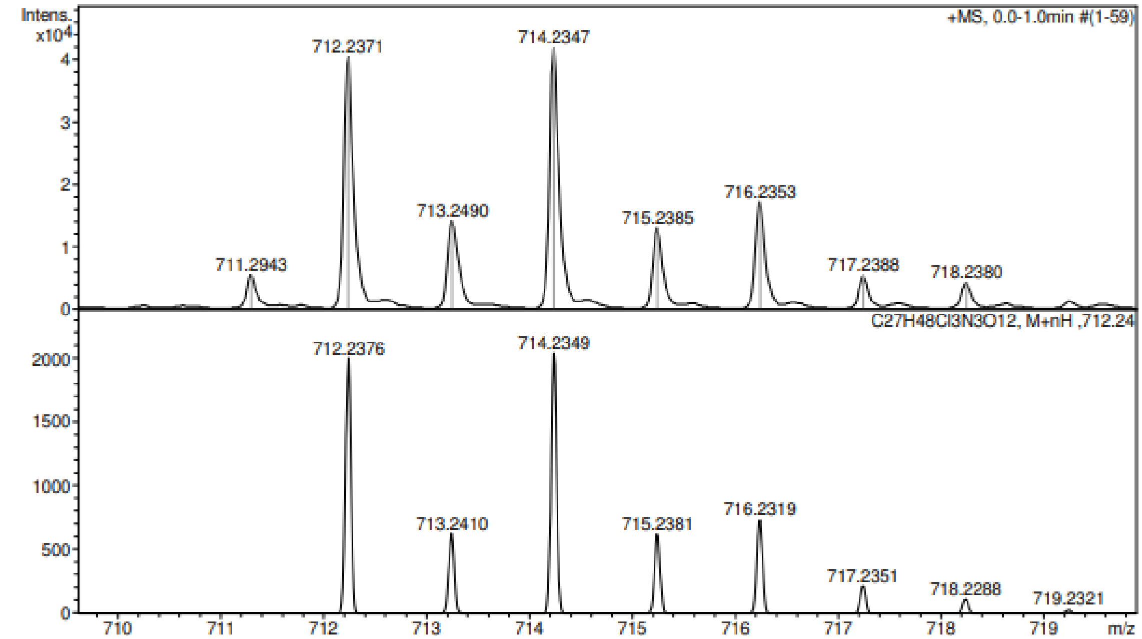

^{1}H, ^{13}C NMR and HRMS spectra of Novel pyridinium tris-QACs

^{1}H NMR spectrum of 1,1',1''-((((2,4,6-trioxo-1,3,5-triazinane-1,3,5-triyl)tris(ethane-2,1-diyl))tris(oxy))tris(ethane-2,1-diyl))tris(4-(hexylamino)pyridin-1-ium) **(7a)**

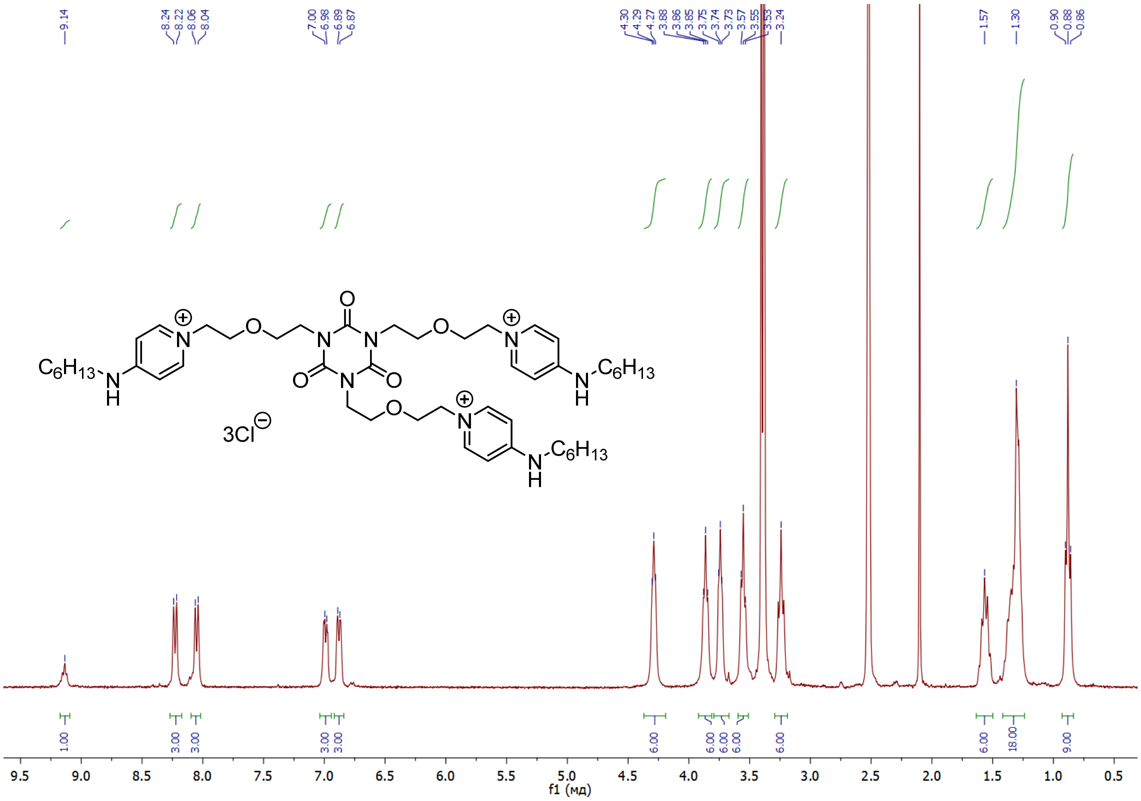

^{13}C NMR spectrum of **7a**

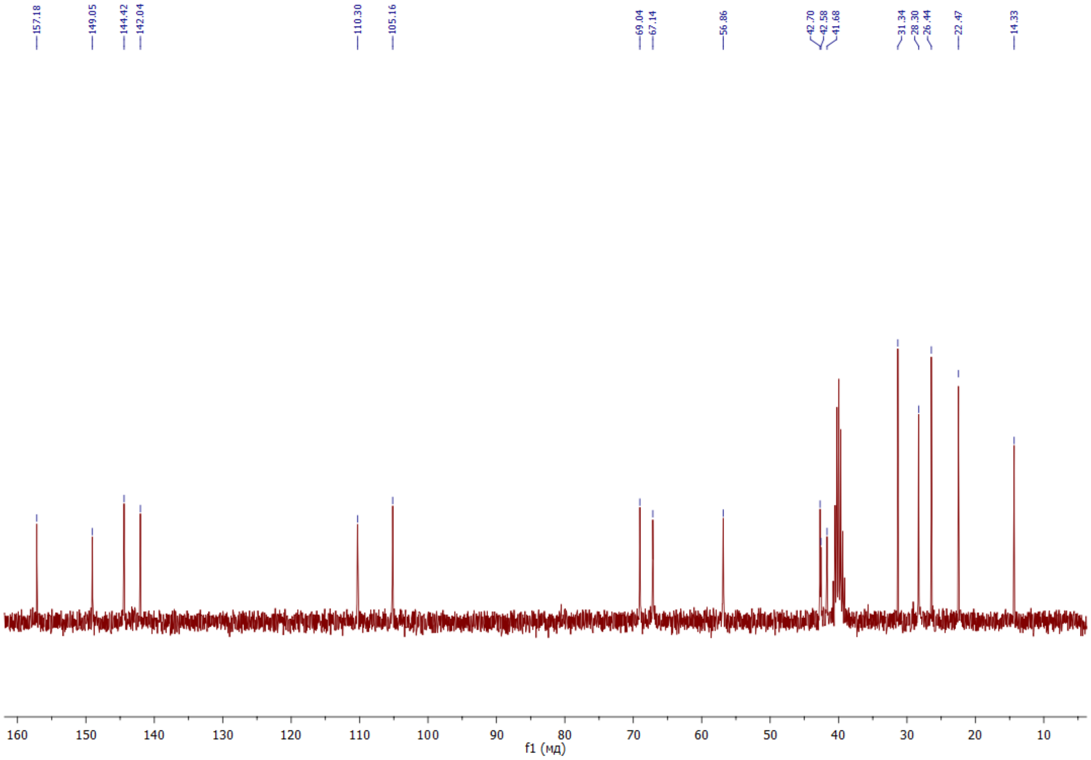

HRMS of 7a

Display Report

Analysis Info

Analysis Name	D:\Data\Kolotyrkina\2023\Seferyan\0426020.d		
Method	tune_wide.m	Operator	BDAL@DE
Sample Name	/VERS MG011.1	Instrument / Ser#	micrOTOF 10248
Comment	C48H78N9O6 mH877.6147 calibrant added CH3OH		

Acquisition Parameter

Source Type	ESI	Ion Polarity	Positive	Set Nebulizer	0.4 Bar
Focus	Not active			Set Dry Heater	180 °C
Scan Begin	50 m/z	Set Capillary	4500 V	Set Dry Gas	4.0 l/min
Scan End	3000 m/z	Set End Plate Offset	-500 V	Set Divert Valve	Waste

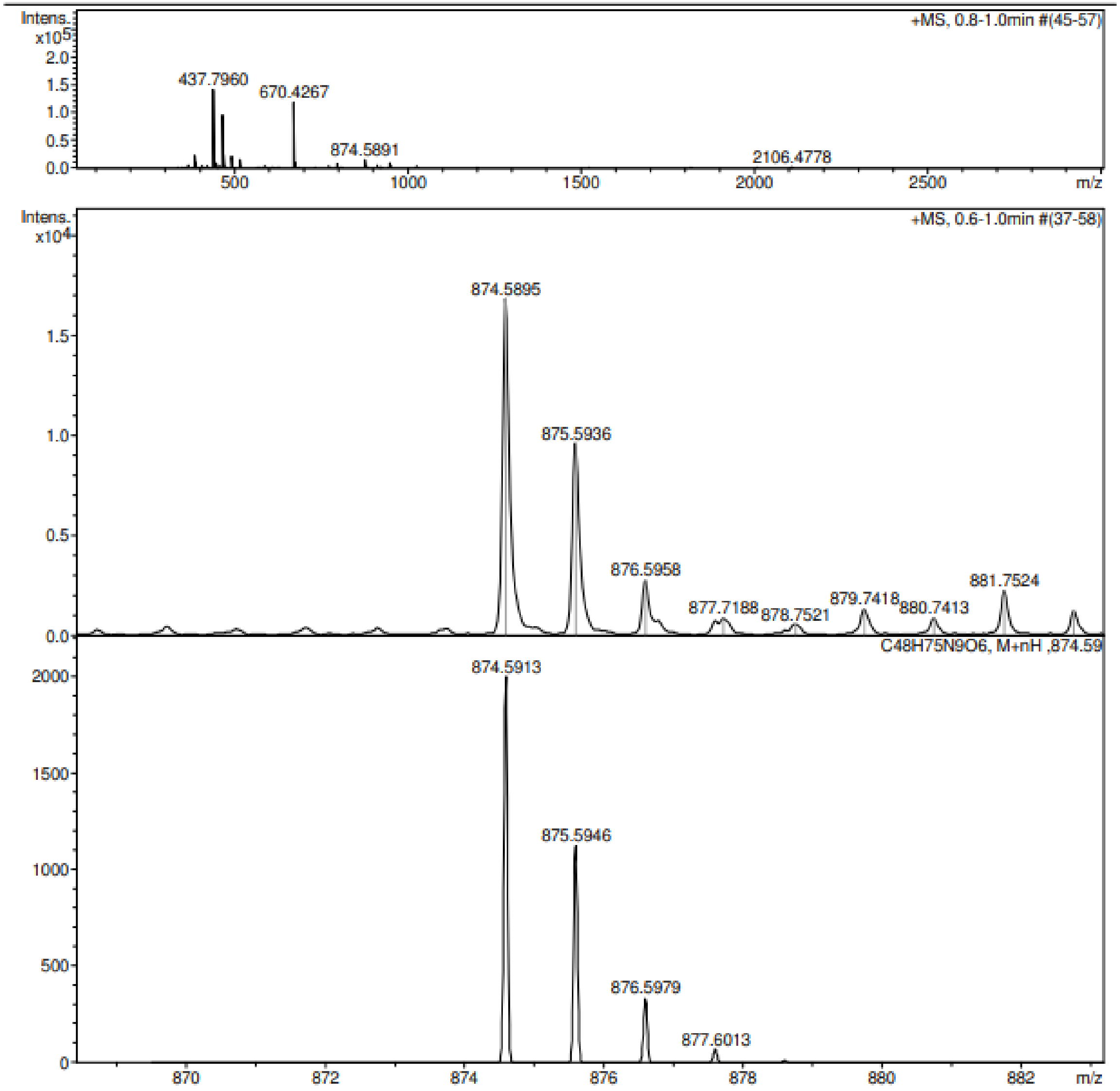

^{1}H NMR spectrum of 1,1',1''-((((2,4,6-trioxo-1,3,5-triazinane-1,3,5-triyl)tris(ethane-2,1-diyl))tris(oxy))tris(ethane-2,1-diyl))tris(4-(heptylamino)pyridin-1-ium) **(7b)**

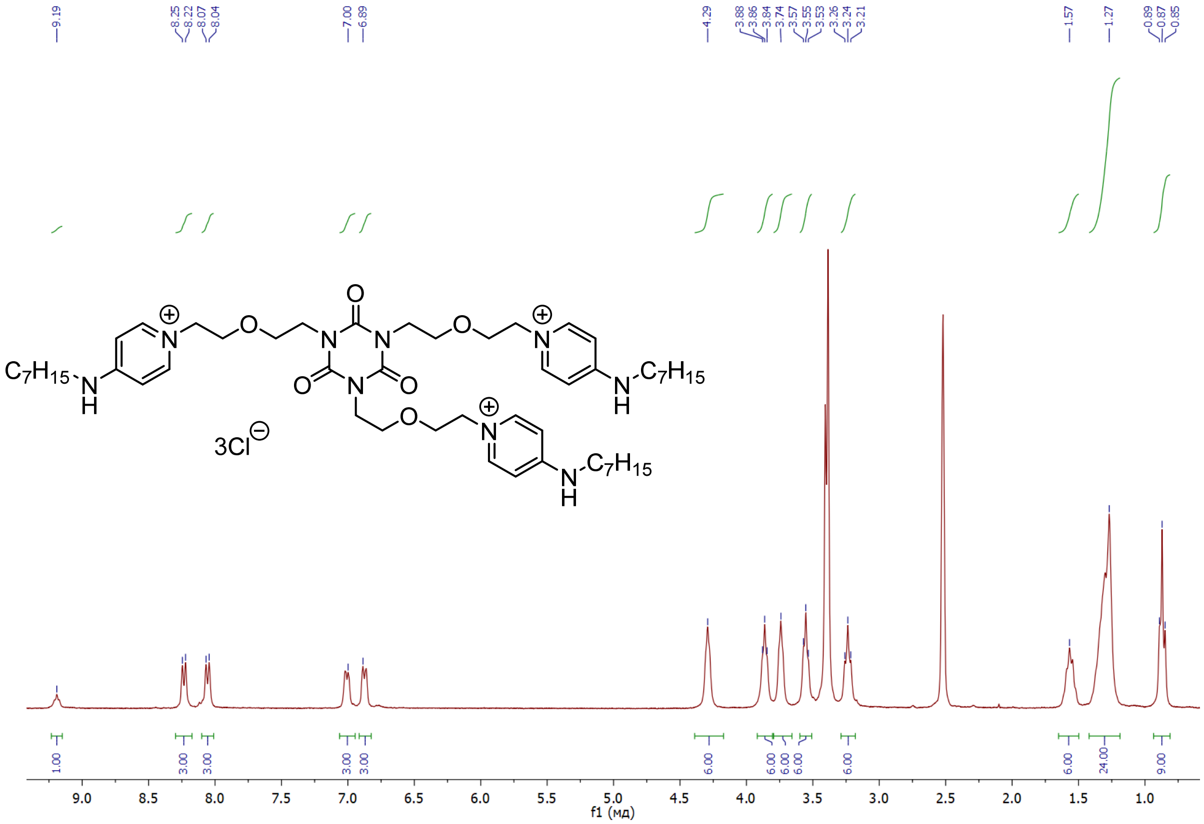

^{13}C NMR spectrum of **7b**

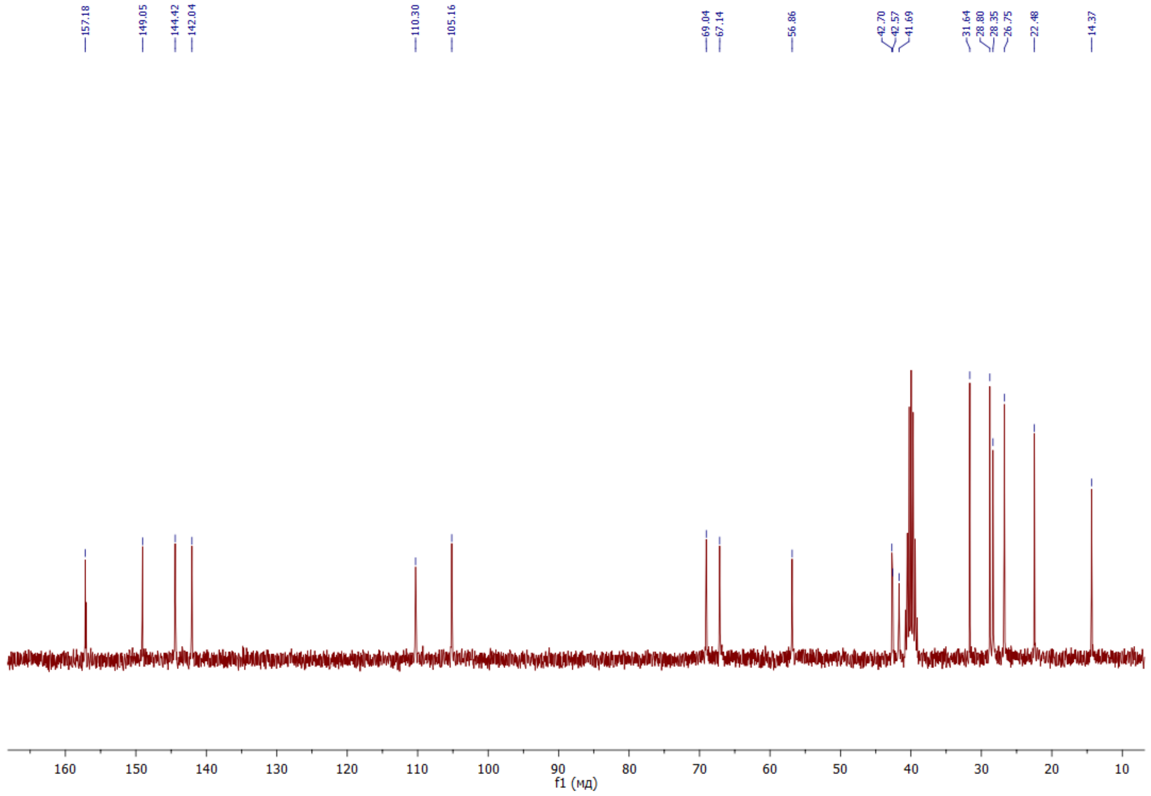

HRMS of 7b

Display Report

Analysis Info

Analysis Name	D:\Data\Kolotyrkina\2023\Seferyan\0426021.d		
Method	tune_wide.m	Operator	BDAL@DE
Sample Name	/VERS MG010.1	Instrument / Ser#	micrOTOF 10248
Comment	C51H81N9O6 mH916.6382 calibrant added CH3OH		

Acquisition Parameter

Source Type	ESI	Ion Polarity	Positive	Set Nebulizer	0.4 Bar
Focus	Not active			Set Dry Heater	180 °C
Scan Begin	50 m/z	Set Capillary	4500 V	Set Dry Gas	4.0 l/min
Scan End	3000 m/z	Set End Plate Offset	-500 V	Set Divert Valve	Waste

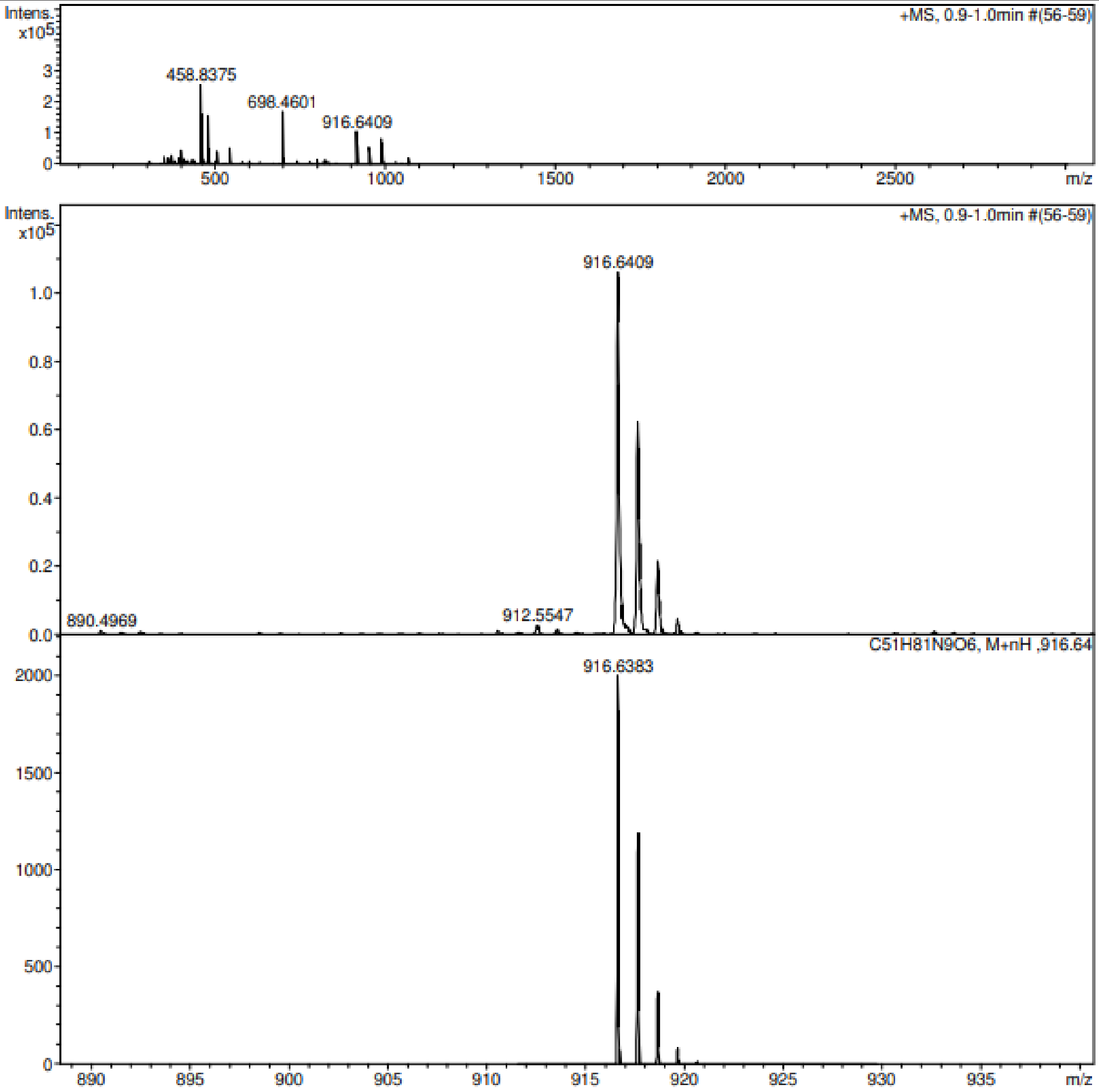

[1]**H NMR** spectrum of 1,1',1''-((((2,4,6-trioxo-1,3,5-triazinane-1,3,5-triyl)tris(ethane-2,1-diyl))tris(oxy))tris(ethane-2,1-diyl))tris(4-(octylamino)pyridin-1-ium) **(7c)**

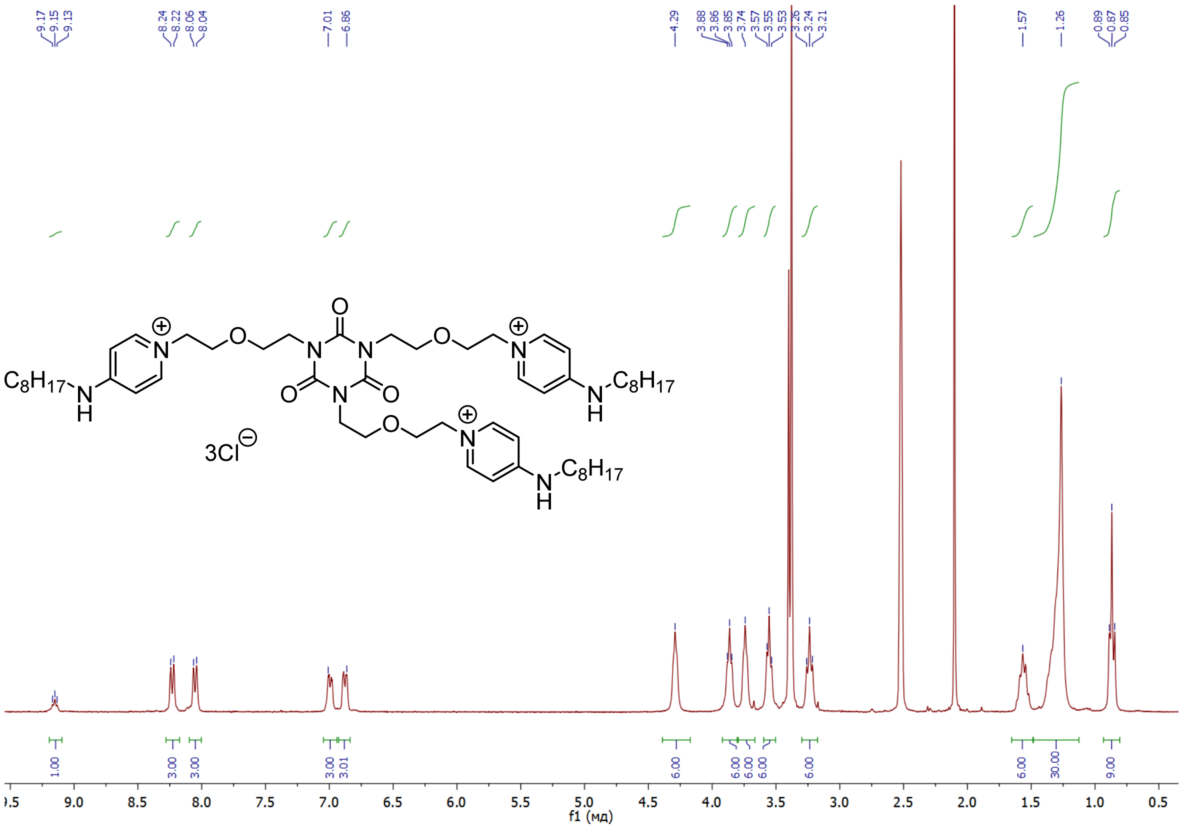

[13]**C NMR** spectrum of **7c**

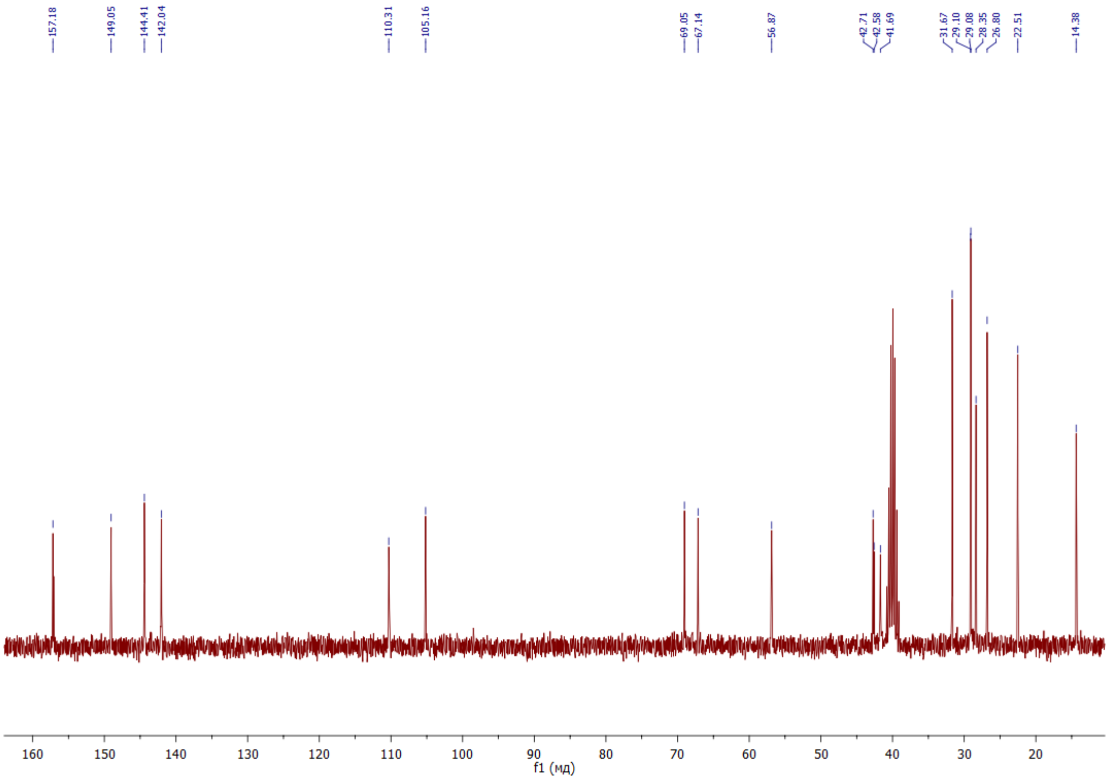

HRMS of 7c

Display Report

Analysis Info

Analysis Name	D:\Data\Kolotyrkina\2023\Seferyan\0426022.d		
Method	tune_wide.m	Operator	BDAL@DE
Sample Name	/VERS MG009.1	Instrument / Ser#	micrOTOF 10248
Comment	C54H87N9O6 mH958.6852 calibrant added CH3OH		

Acquisition Parameter

Source Type	ESI	Ion Polarity	Positive	Set Nebulizer	0.4 Bar
Focus	Not active			Set Dry Heater	180 °C
Scan Begin	50 m/z	Set Capillary	4500 V	Set Dry Gas	4.0 l/min
Scan End	3000 m/z	Set End Plate Offset	-500 V	Set Divert Valve	Waste

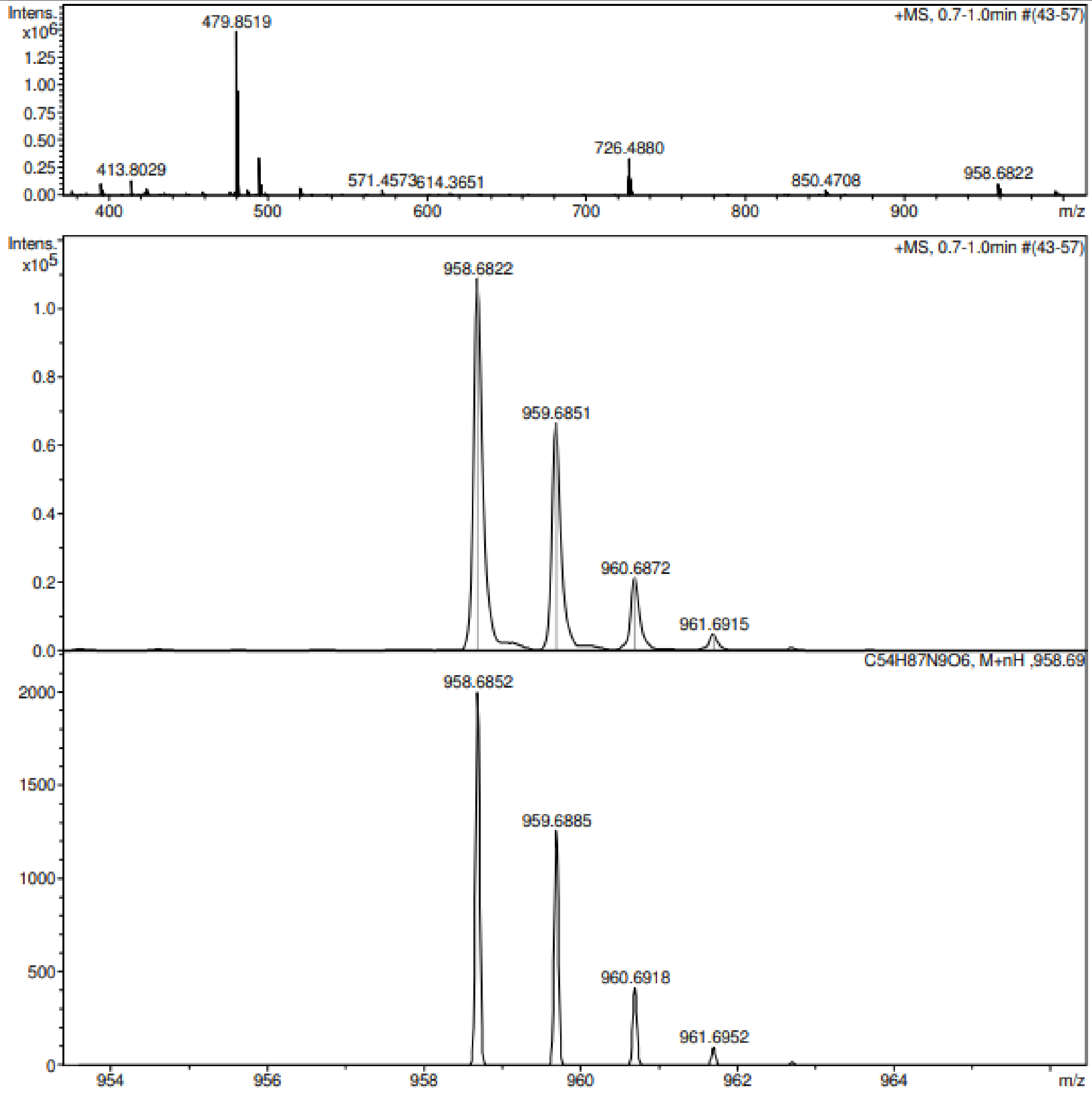

[1]**H NMR** spectrum of 1,1',1''-((((2,4,6-trioxo-1,3,5-triazinane-1,3,5-triyl)tris(ethane-2,1-diyl))tris(oxy))tris(ethane-2,1-diyl))tris(4-(nonylamino)pyridin-1-ium) **(7d)**

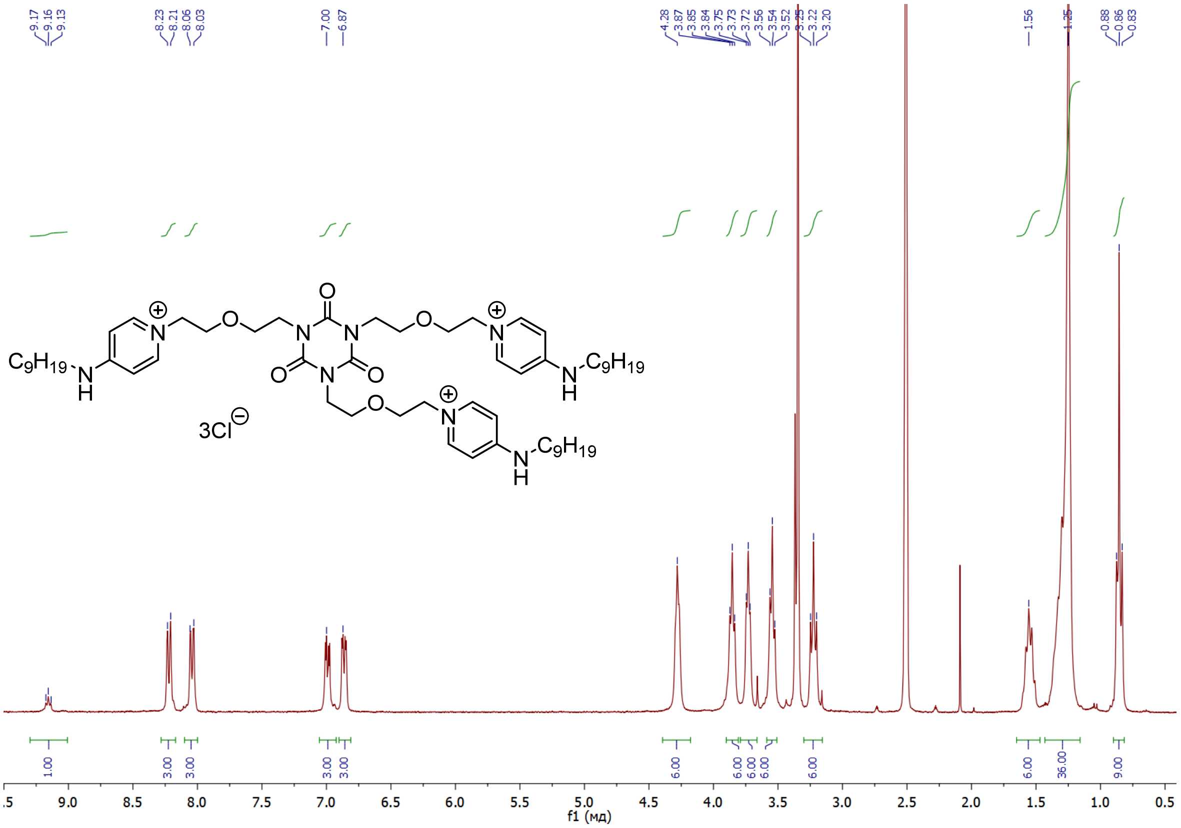

[13]**C NMR** spectrum of **7d**

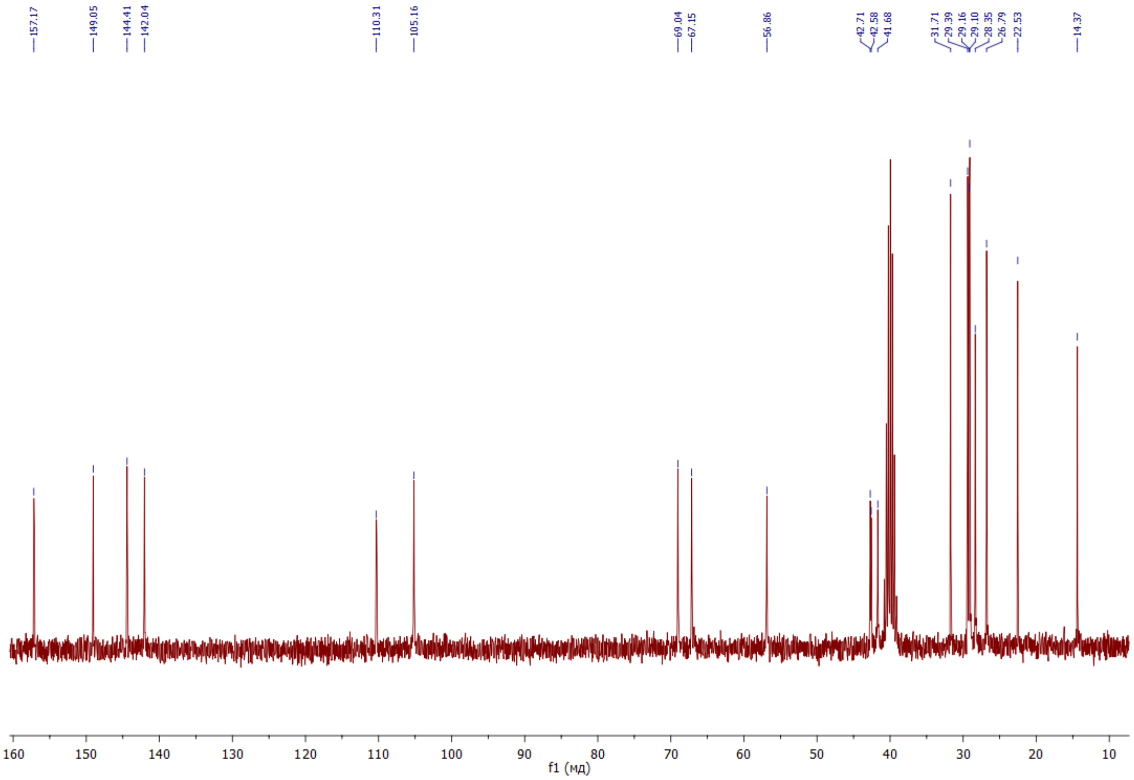

HRMS of 7d

Display Report

Analysis Info

Analysis Name	D:\Data\Kolotyrkina\2023\Seferyan\0426023.d		
Method	tune_wide.m	Operator	BDAL@DE
Sample Name	/VERS MG002.1	Instrument / Ser#	micrOTOF 10248
Comment	C57H93N9O6 mH100.7321 calibrant added CH3OH		

Acquisition Parameter

Source Type	ESI	Ion Polarity	Positive	Set Nebulizer	0.4 Bar
Focus	Not active			Set Dry Heater	180 °C
Scan Begin	50 m/z	Set Capillary	4500 V	Set Dry Gas	4.0 l/min
Scan End	3000 m/z	Set End Plate Offset	-500 V	Set Divert Valve	Waste

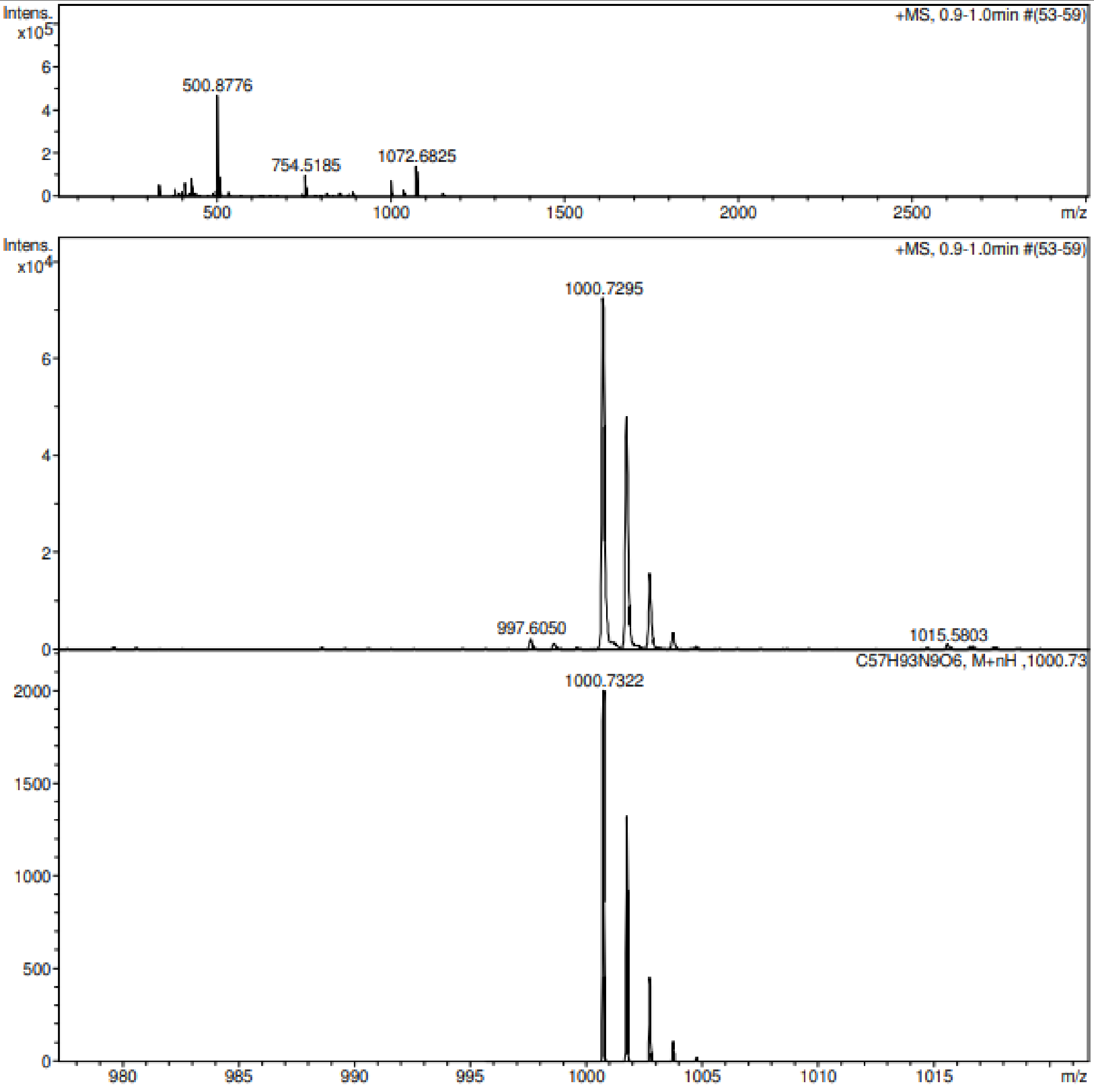

^{1}H NMR spectrum of 1,1',1''-((((2,4,6-trioxo-1,3,5-triazinane-1,3,5-triyl)tris(ethane-2,1-diyl))tris(oxy))tris(ethane-2,1-diyl))tris(4-(decylamino)pyridin-1-ium) **(7e)**

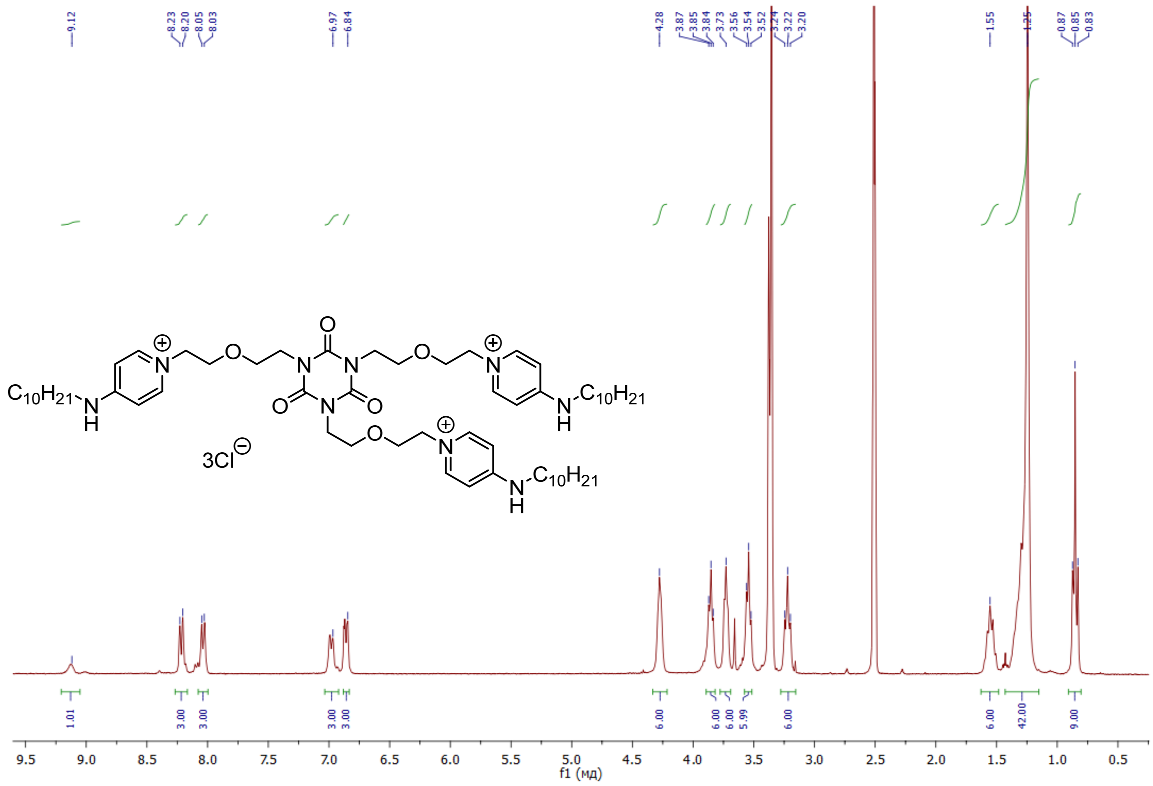

^{13}C NMR spectrum of **7e**

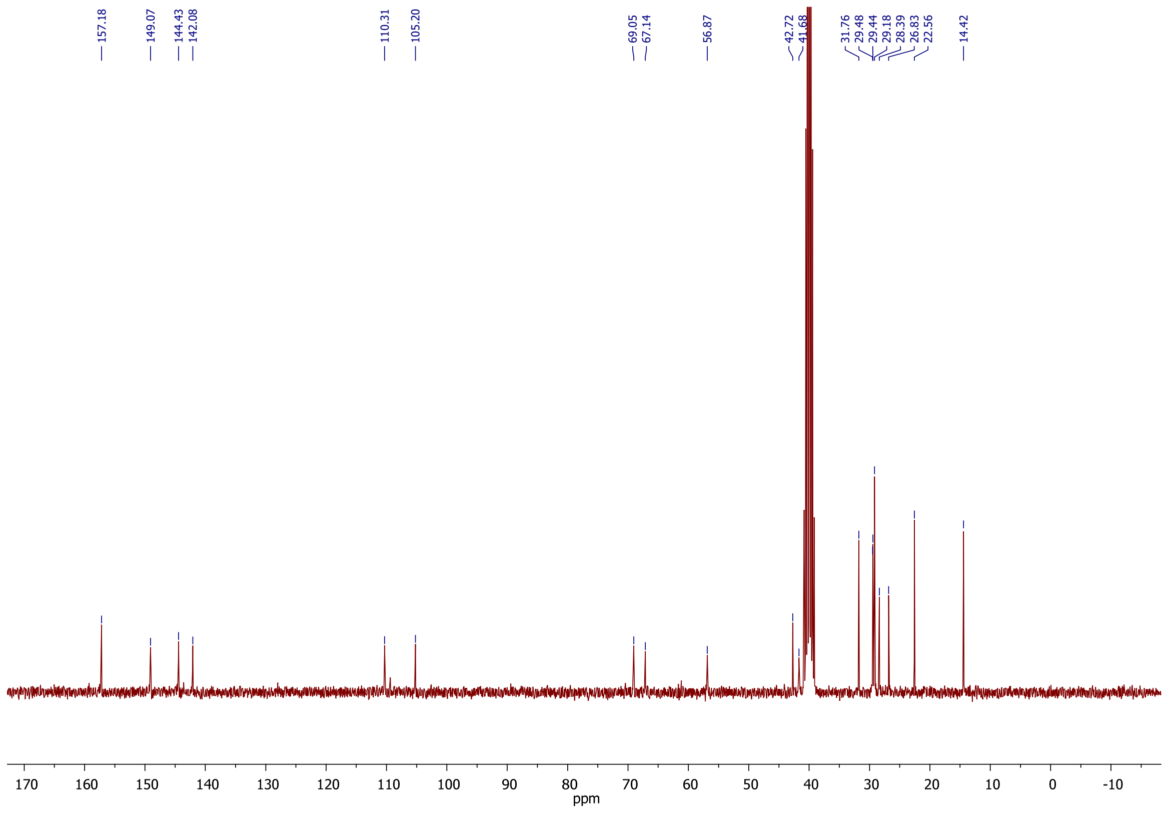

[1]**H NMR** spectrum of 1,1',1''-((((2,4,6-trioxo-1,3,5-triazinane-1,3,5-triyl)tris(ethane-2,1-diyl))tris(oxy))tris(ethane-2,1-diyl))tris(4-(undecylamino)pyridin-1-ium) **(7f)**

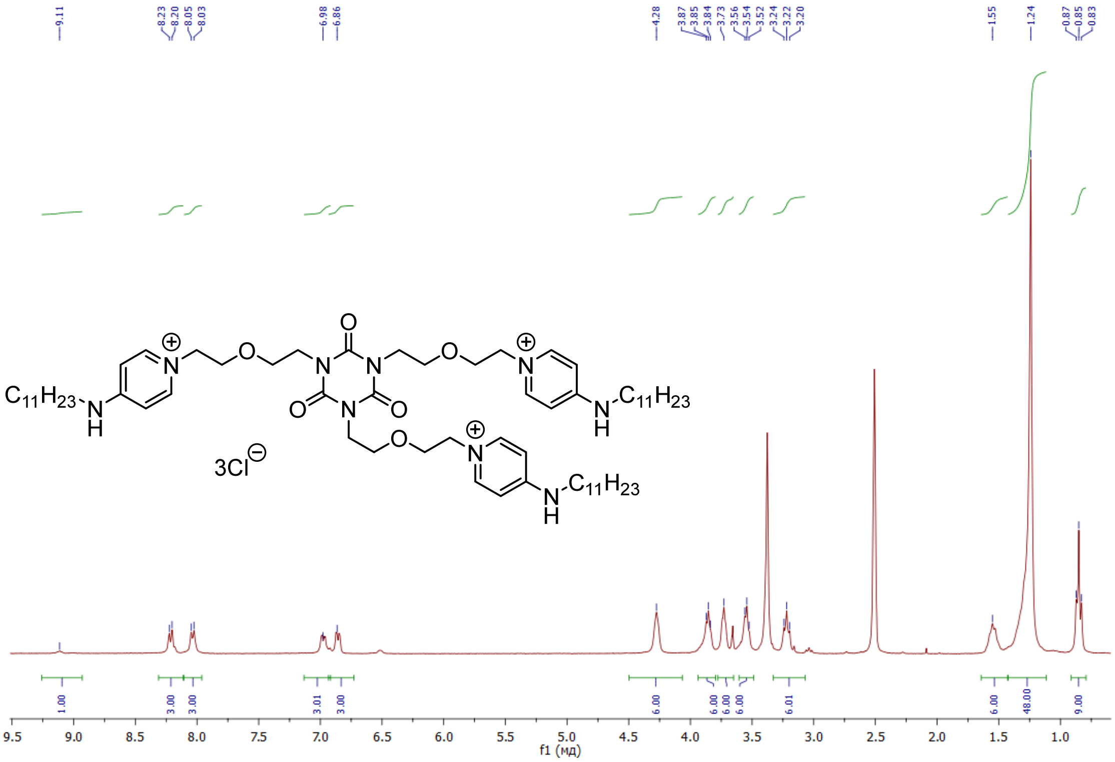

[13]**C NMR** spectrum of **7f**

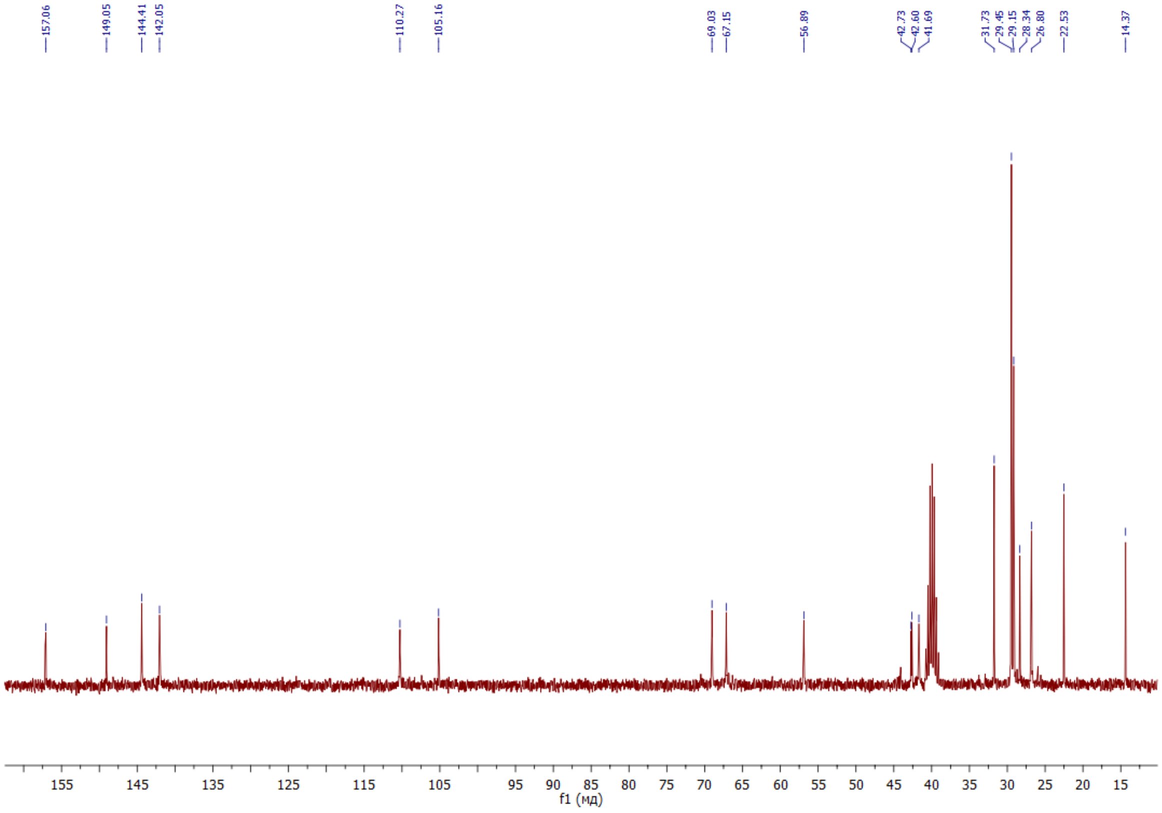

HRMS of 7f

Display Report

Analysis Info

Analysis Name	D:\Data\Kolotyrkina\2023\Seferyan\0426025.d		
Method	tune_wide.m	Operator	BDAL@DE
Sample Name	/VERS MV009.21	Instrument / Ser#	micrOTOF 10248
Comment	C63H105N9O6 mH1084.8260 calibrant added CH3OH		

Acquisition Parameter

Source Type	ESI	Ion Polarity	Positive	Set Nebulizer	0.4 Bar
Focus	Not active			Set Dry Heater	180 °C
Scan Begin	50 m/z	Set Capillary	4500 V	Set Dry Gas	4.0 l/min
Scan End	3000 m/z	Set End Plate Offset	-500 V	Set Divert Valve	Waste

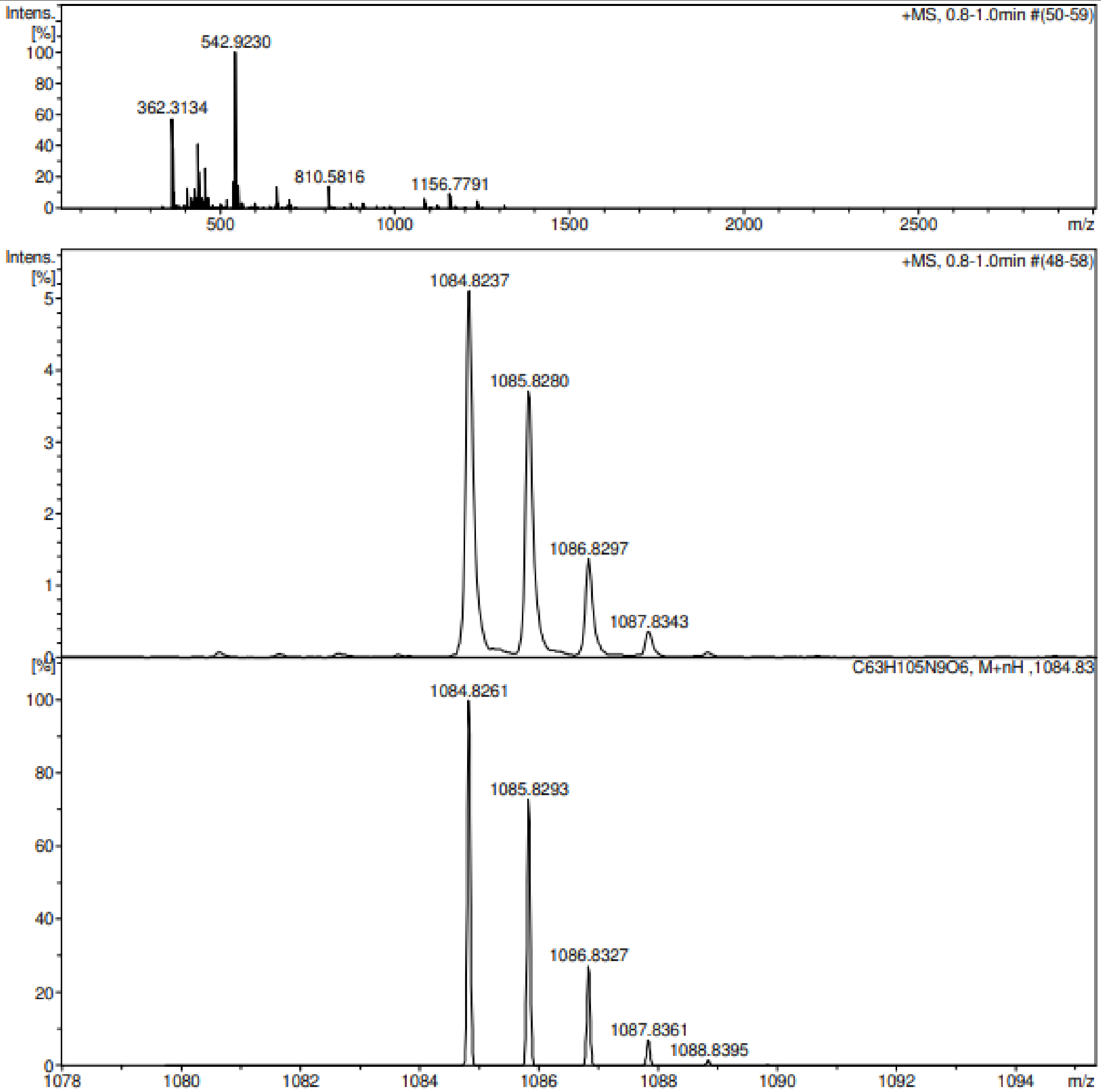

¹H NMR spectrum of 1,1',1"-((((2,4,6-trioxo-1,3,5-triazinane-1,3,5-triyl)tris(ethane-2,1-diyl))tris(oxy))tris(ethane-2,1-diyl))tris(4-(dodecylamino)pyridin-1-ium) **(7g)**

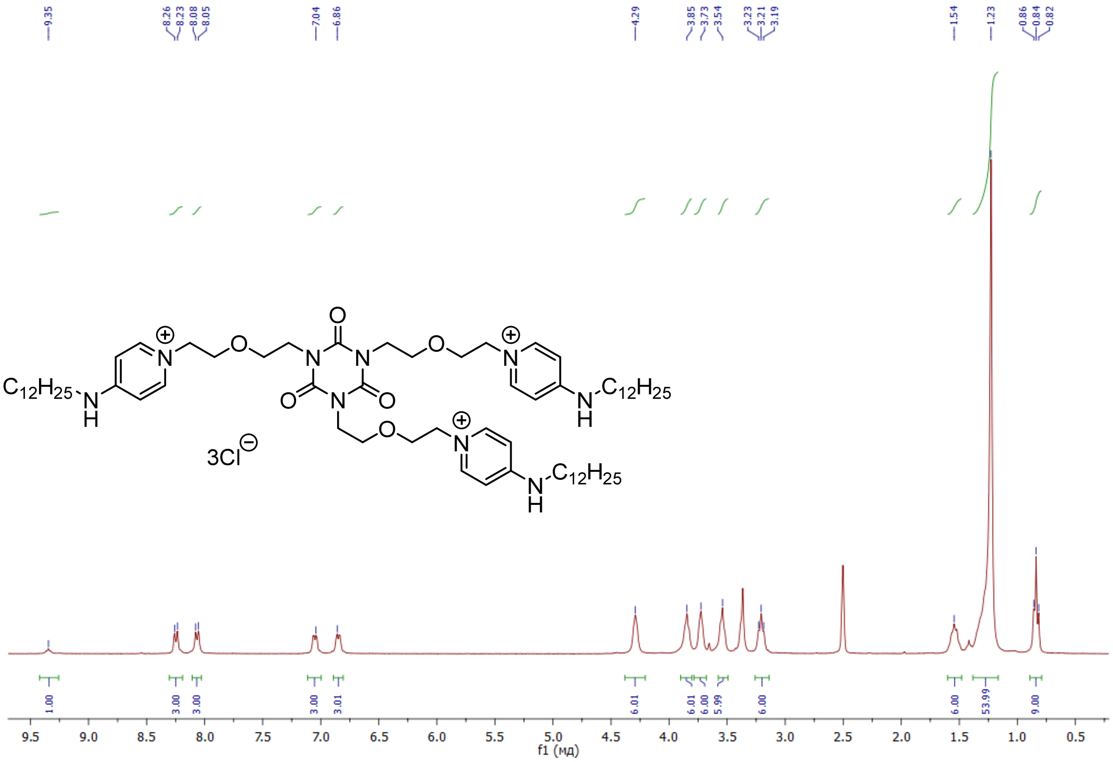

¹³C NMR spectrum of **7g**

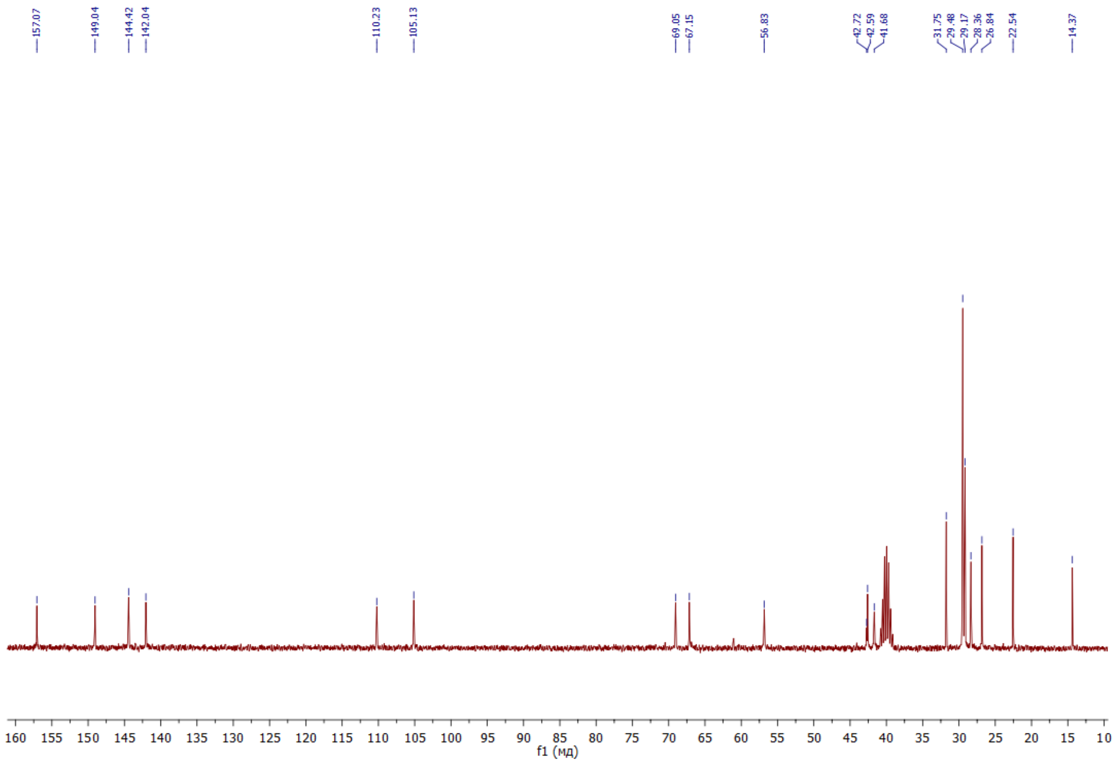

HRMS of 7g

Display Report

Analysis Info

Analysis Name	D:\Data\Kolotyrkina\2023\Seferyan\0426024.d		
Method	tune_wide.m	Operator	BDAL@DE
Sample Name	/VERS TA018.1	Instrument / Ser#	micrOTOF 10248
Comment	C66H111N9O6 mH1126.8730 calibrant added CH3OH		

Acquisition Parameter

Source Type	ESI	Ion Polarity	Positive	Set Nebulizer	0.4 Bar
Focus	Not active			Set Dry Heater	180 °C
Scan Begin	50 m/z	Set Capillary	4500 V	Set Dry Gas	4.0 l/min
Scan End	3000 m/z	Set End Plate Offset	-500 V	Set Divert Valve	Waste

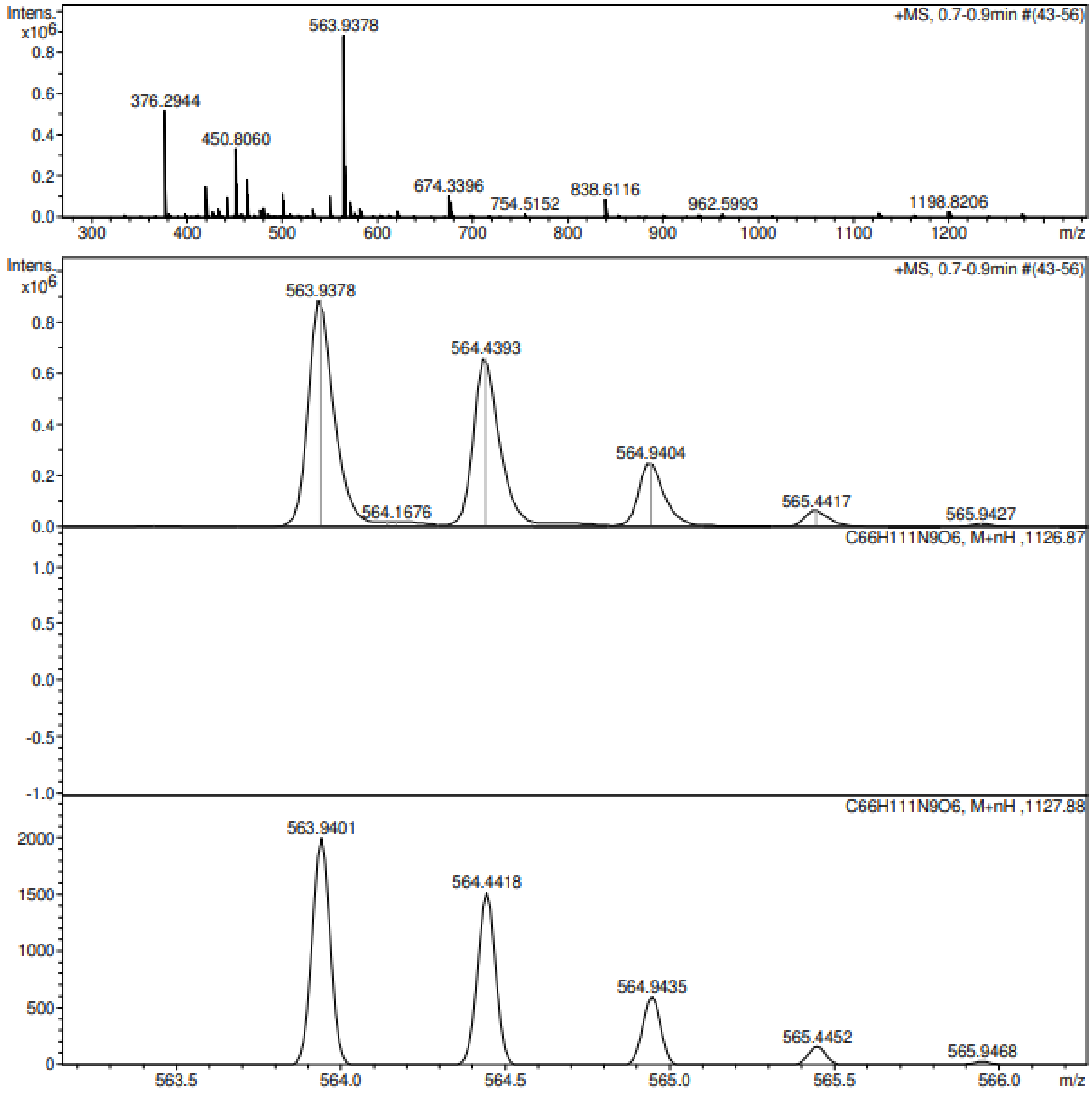

^{1}H NMR spectrum of 1,1',1''-((((((2,4,6-trioxo-1,3,5-triazinane-1,3,5-triyl)tris(ethane-2,1-diyl))tris(oxy))tris(ethane-2,1-diyl))tris(oxy))tris(ethane-2,1-diyl))tris(4-(octylamino)pyridin-1-ium) **(8a)**

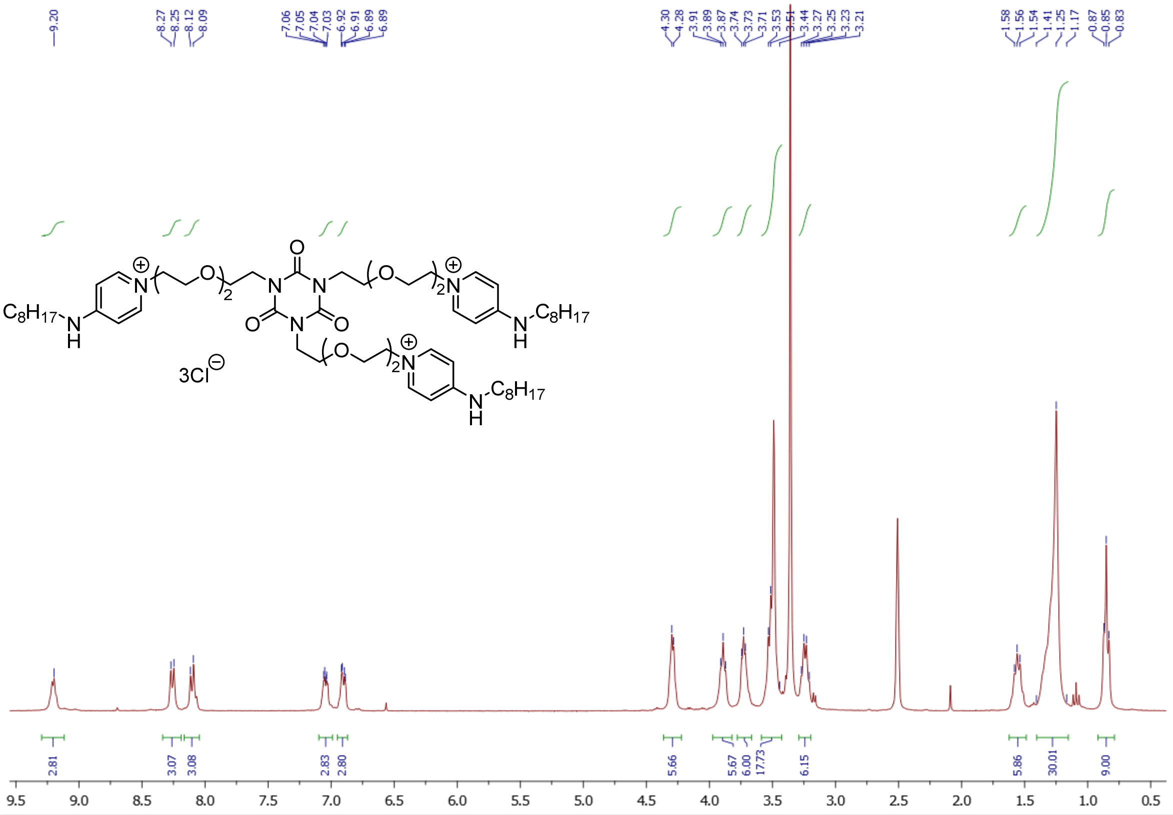

^{13}C NMR spectrum of **8a**

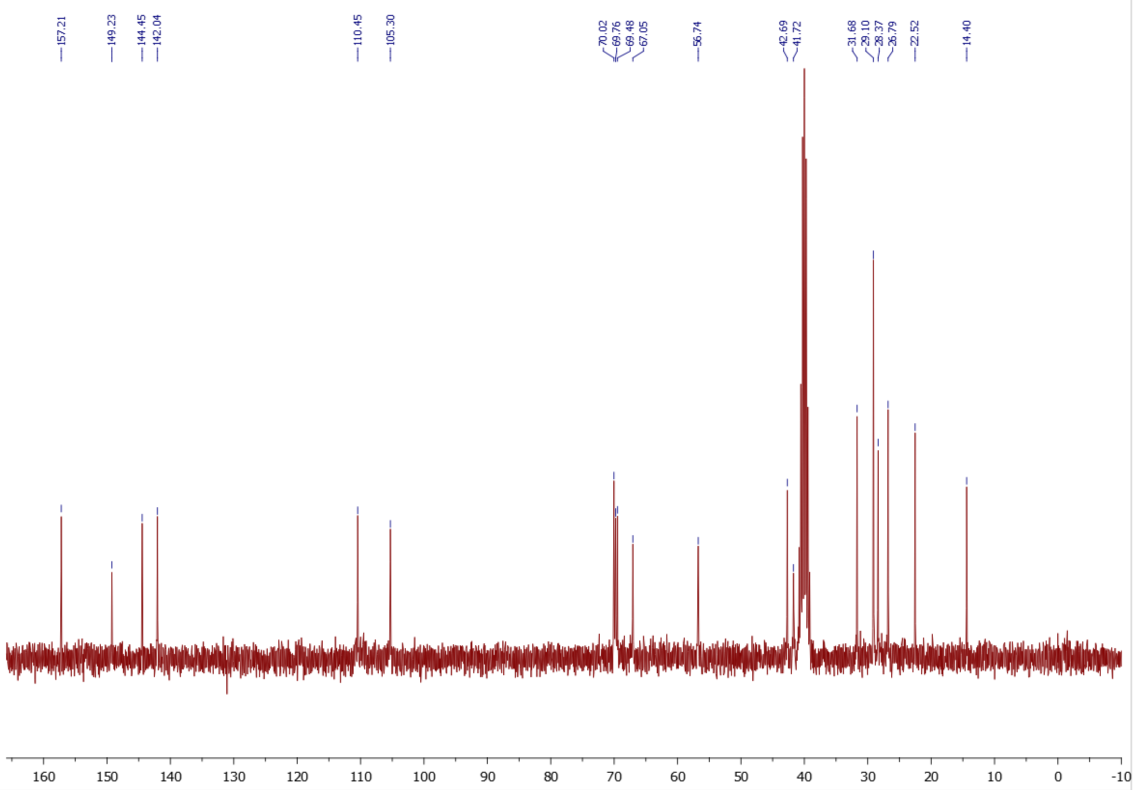

HRMS of **8a**

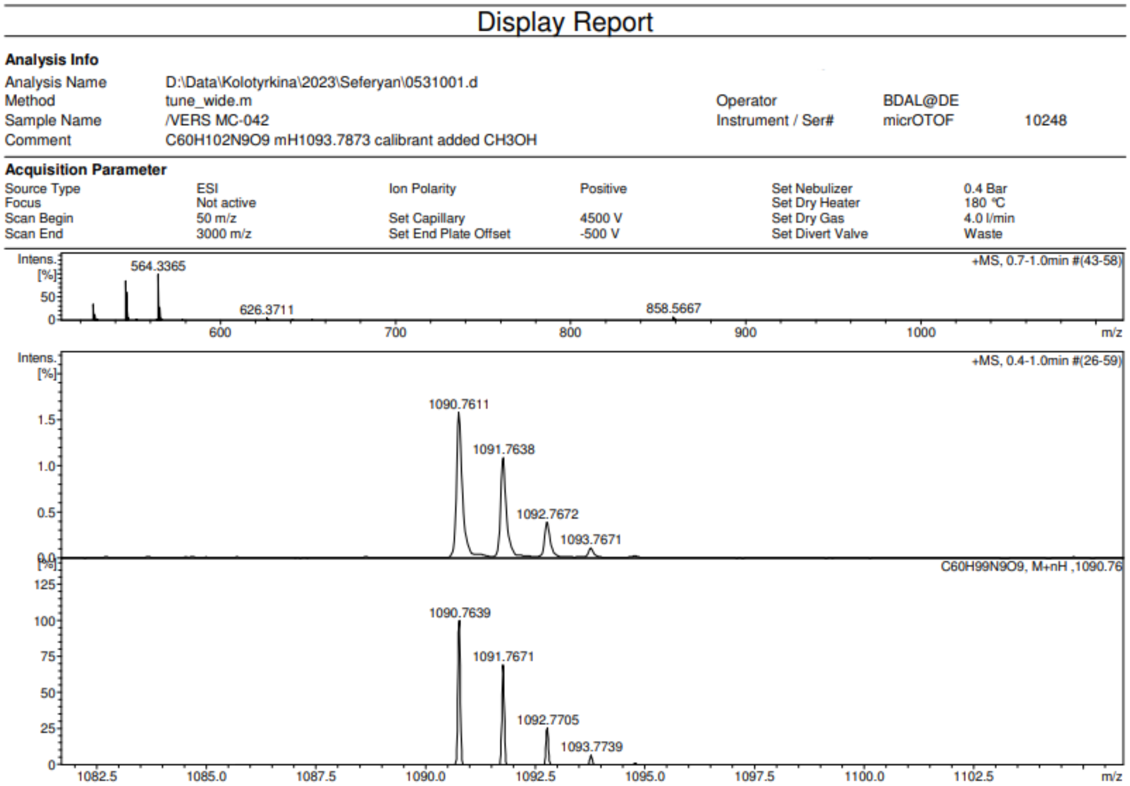
Display Report
Analysis Info
Analysis Name D:\Data\Kolotyrkina\2023\Seferyan\0531001.d
Method tune_wide.m
Sample Name /VERS MC-042
Comment C60H102N9O9 mH1093.7873 calibrant added CH3OH
Operator BDAL@DE
Instrument / Ser# micrOTOF 10248
Acquisition Parameter
Source Type ESI
Focus Not active
Scan Begin 50 m/z
Scan End 3000 m/z
Ion Polarity Positive
Set Capillary 4500 V
Set End Plate Offset -500 V
Set Nebulizer 0.4 Bar
Set Dry Heater 180 °C
Set Dry Gas 4.0 l/min
Set Divert Valve Waste
Intens. [%]
564.3365
626.3711
858.5667
+MS, 0.7-1.0min #(43-58)
m/z
+MS, 0.4-1.0min #(26-59)
1090.7611
1091.7638
1092.7672
1093.7671
C60H99N9O9, M+nH ,1090.76
1090.7639
1091.7671
1092.7705
1093.7739

^{1}H NMR spectrum of 1,1',1"-((((((2,4,6-trioxo-1,3,5-triazinane-1,3,5-triyl)tris(ethane-2,1-diyl))tris(oxy))tris(ethane-2,1-diyl))tris(oxy))tris(ethane-2,1-diyl))tris(4-(octylamino)pyridin-1-ium) **(8b)**

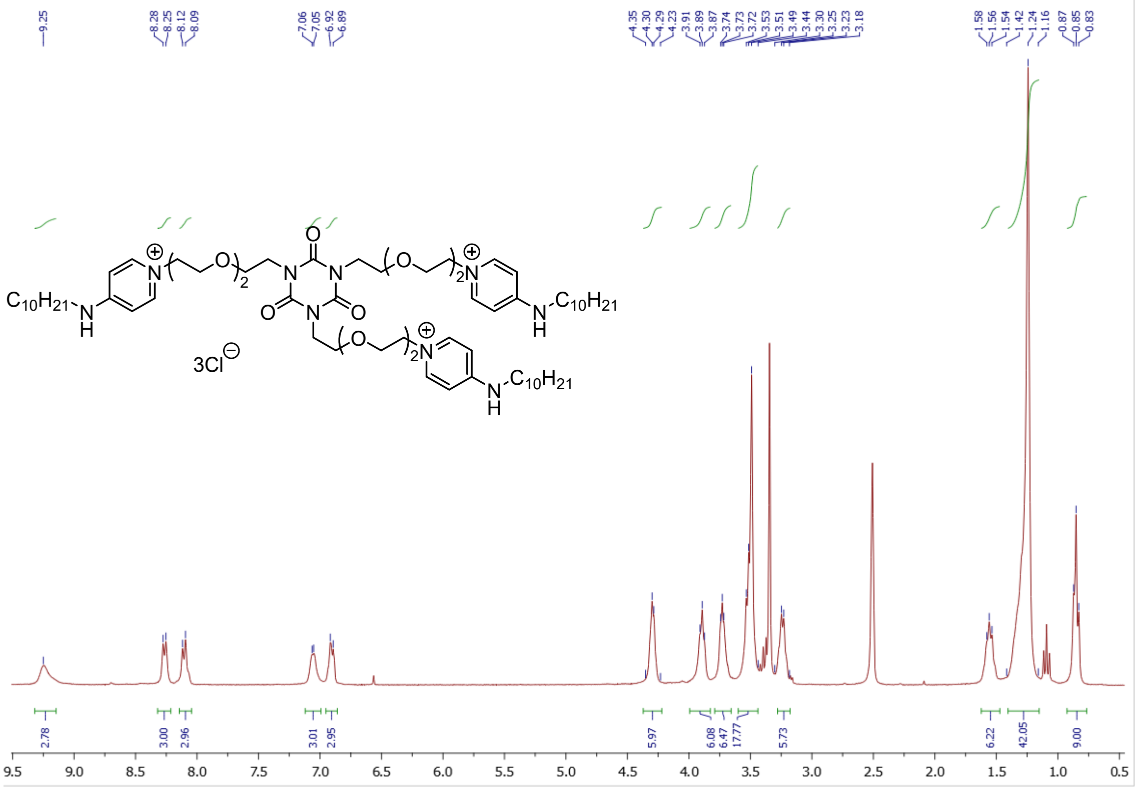

^{13}C NMR spectrum of **8b**

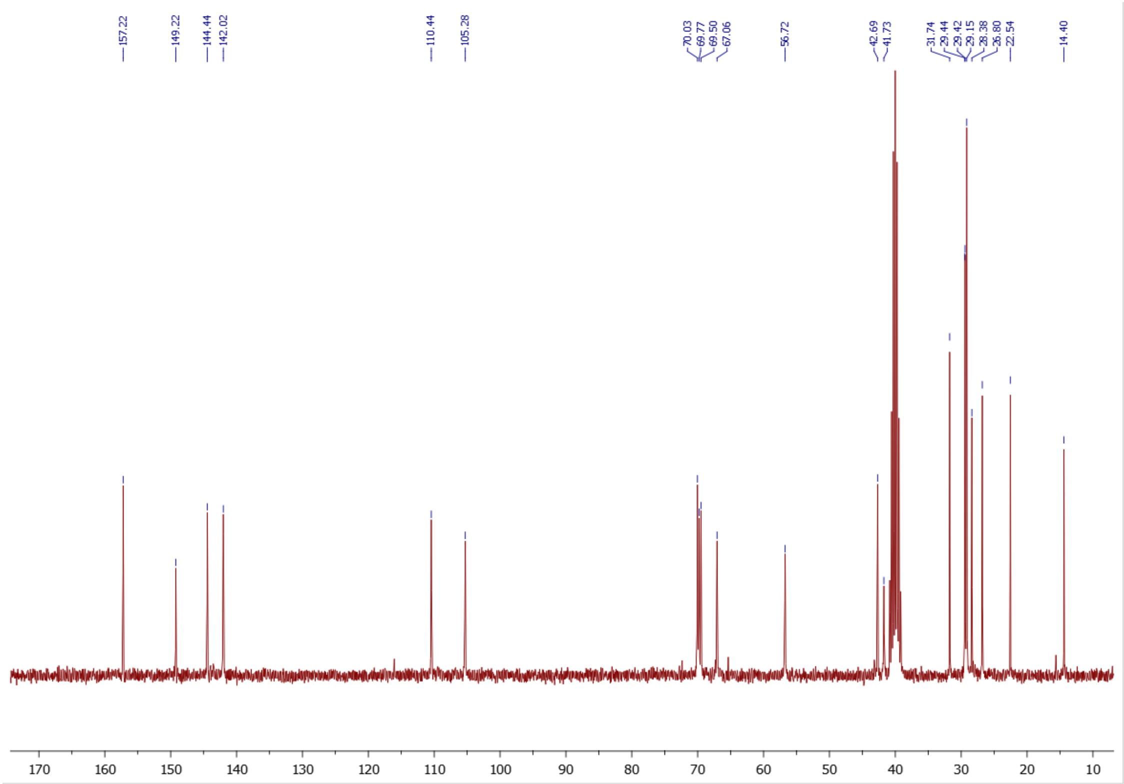

HRMS of **8b**

Display Report

Analysis Info

Analysis Name	D:\Data\Kolotyrkina\2023\Seferyan\0531002.d		
Method	tune_wide.m	Operator	BDAL@DE
Sample Name	/VERS MC-043	Instrument / Ser#	micrOTOF 10248
Comment	C66H114N9O9 mH1177.8812 calibrant added CH3OH		

Acquisition Parameter

Source Type	ESI	Ion Polarity	Positive	Set Nebulizer	0.4 Bar
Focus	Not active			Set Dry Heater	180 °C
Scan Begin	50 m/z	Set Capillary	4500 V	Set Dry Gas	4.0 l/min
Scan End	3000 m/z	Set End Plate Offset	-500 V	Set Divert Valve	Waste

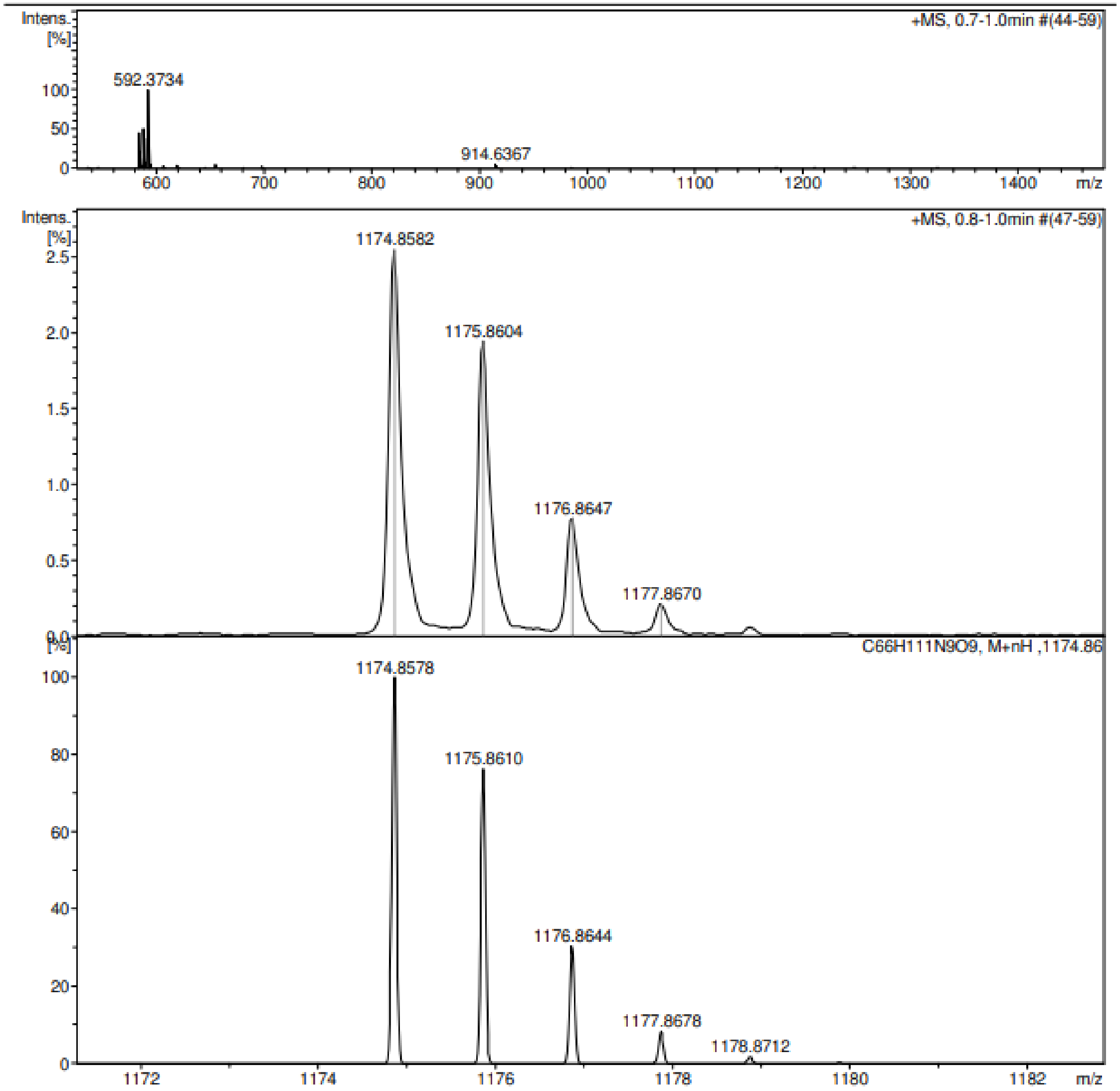

^{1}H NMR 1,1',1''-((((((2,4,6-trioxo-1,3,5-triazinane-1,3,5-triyl)tris(ethane-2,1-diyl))tris(oxy))tris(ethane-2,1-diyl))tris(oxy))tris(ethane-2,1-diyl))tris(4-(dodecylamino)pyridin-1-ium) **(8c)**

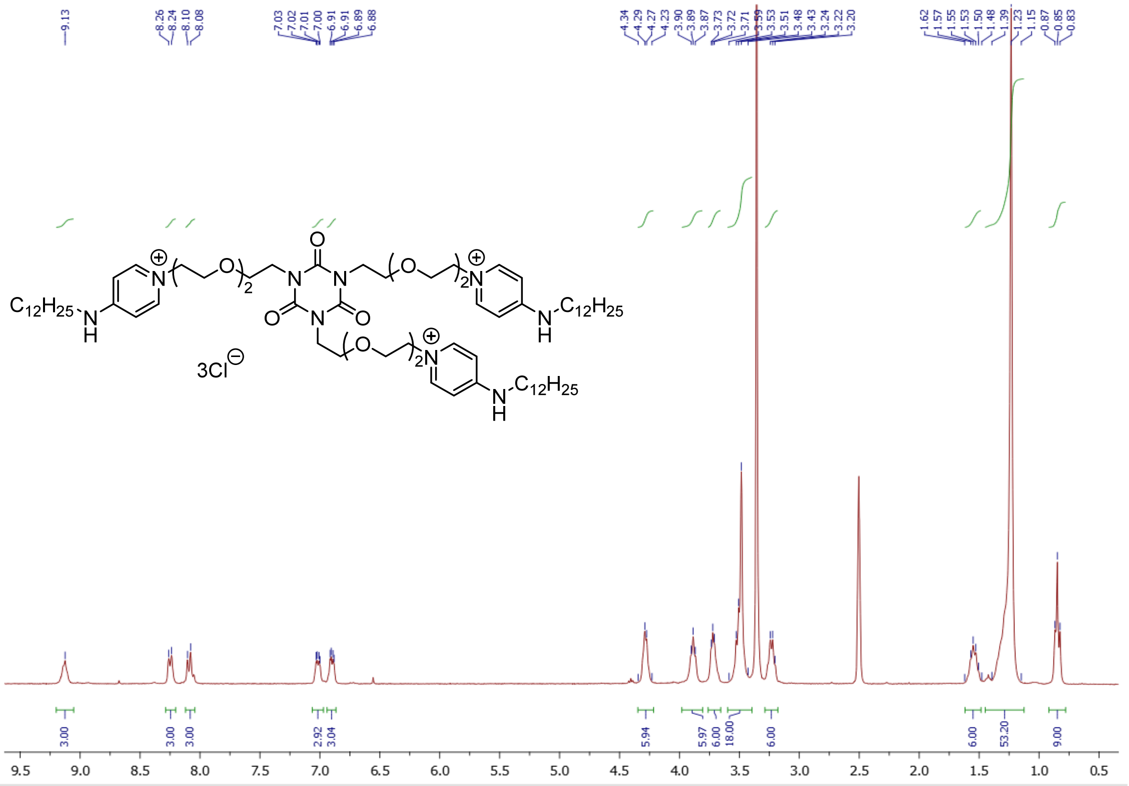

^{13}C NMR spectrum of **8c**

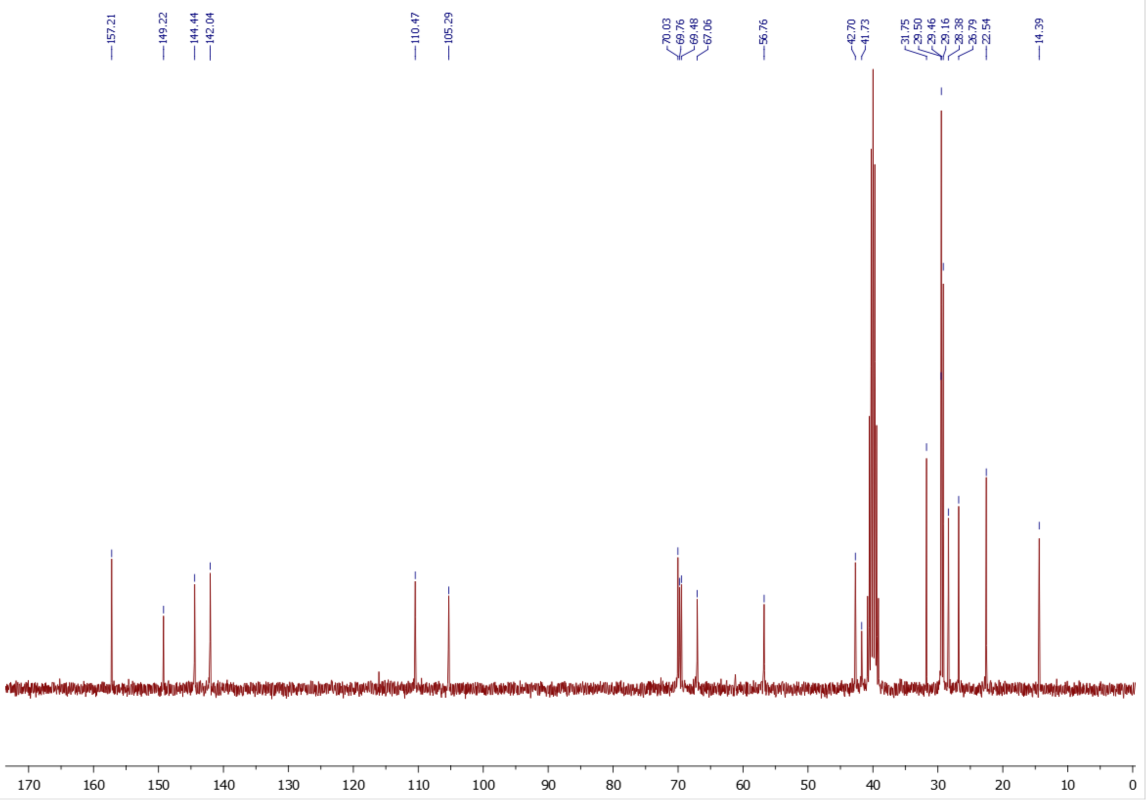

HRMS of **8c**

Display Report

Analysis Info

Analysis Name	D:\Data\Kolotyrkina\2023\Seferyan\0531003.d		
Method	tune_wide.m	Operator	BDAL@DE
Sample Name	/VERS MC-044	Instrument / Ser#	micrOTOF 10248
Comment	C72H126N9O9 mH1261.9751 calibrant added CH3OH		

Acquisition Parameter

Source Type	ESI	Ion Polarity	Positive	Set Nebulizer	0.4 Bar
Focus	Not active			Set Dry Heater	180 °C
Scan Begin	50 m/z	Set Capillary	4500 V	Set Dry Gas	4.0 l/min
Scan End	3000 m/z	Set End Plate Offset	-500 V	Set Divert Valve	Waste

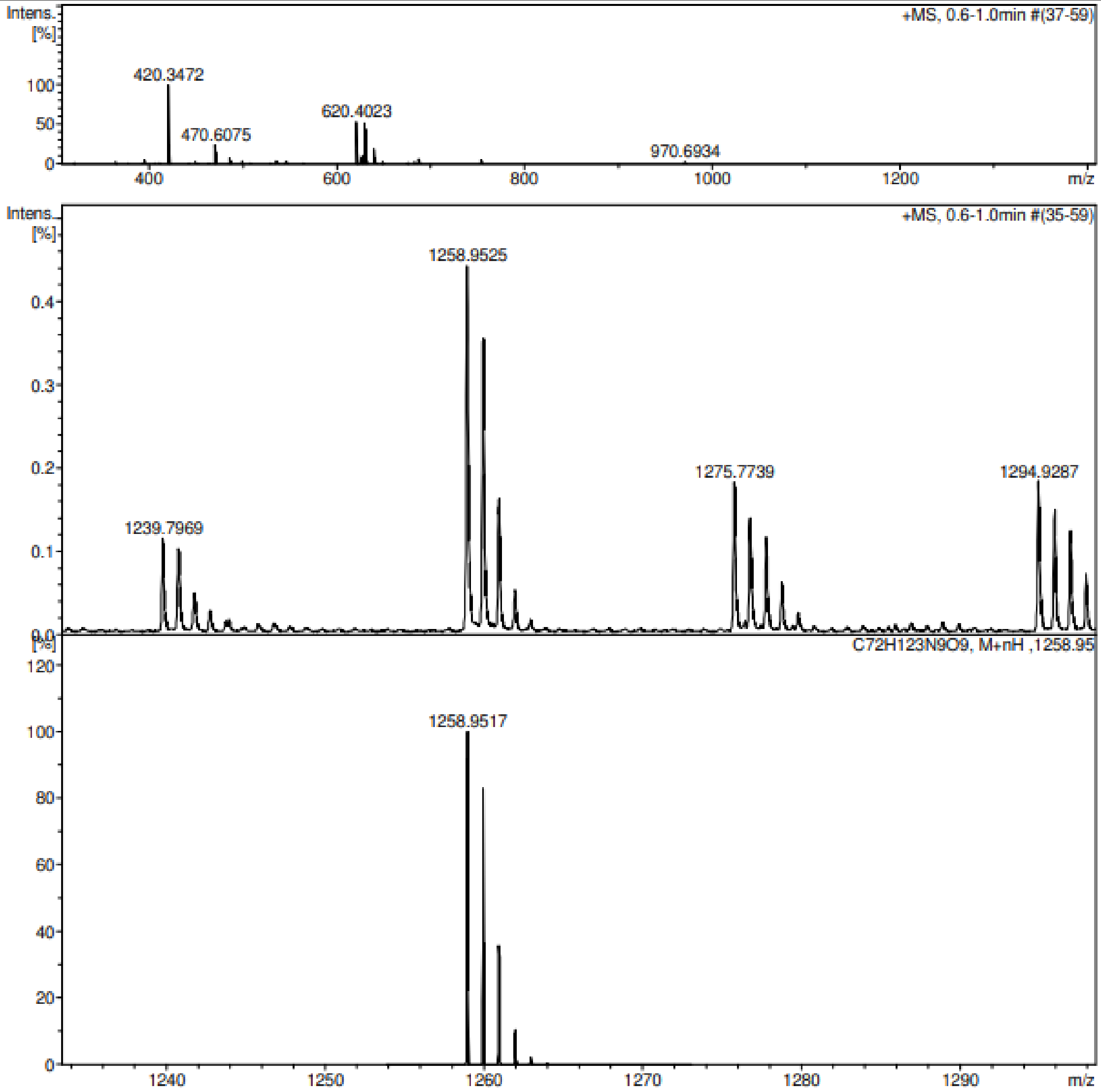

^{1}H NMR spectrum of 1,1',1''-((((((((2,4,6-trioxo-1,3,5-triazinane-1,3,5-triyl)tris(ethane-2,1-diyl))tris(oxy))tris(ethane-2,1-diyl))tris(oxy))tris(ethane-2,1-diyl))tris(oxy))tris(ethane-2,1-diyl))tris(4-(octylamino)pyridin-1-ium) **(9a)**

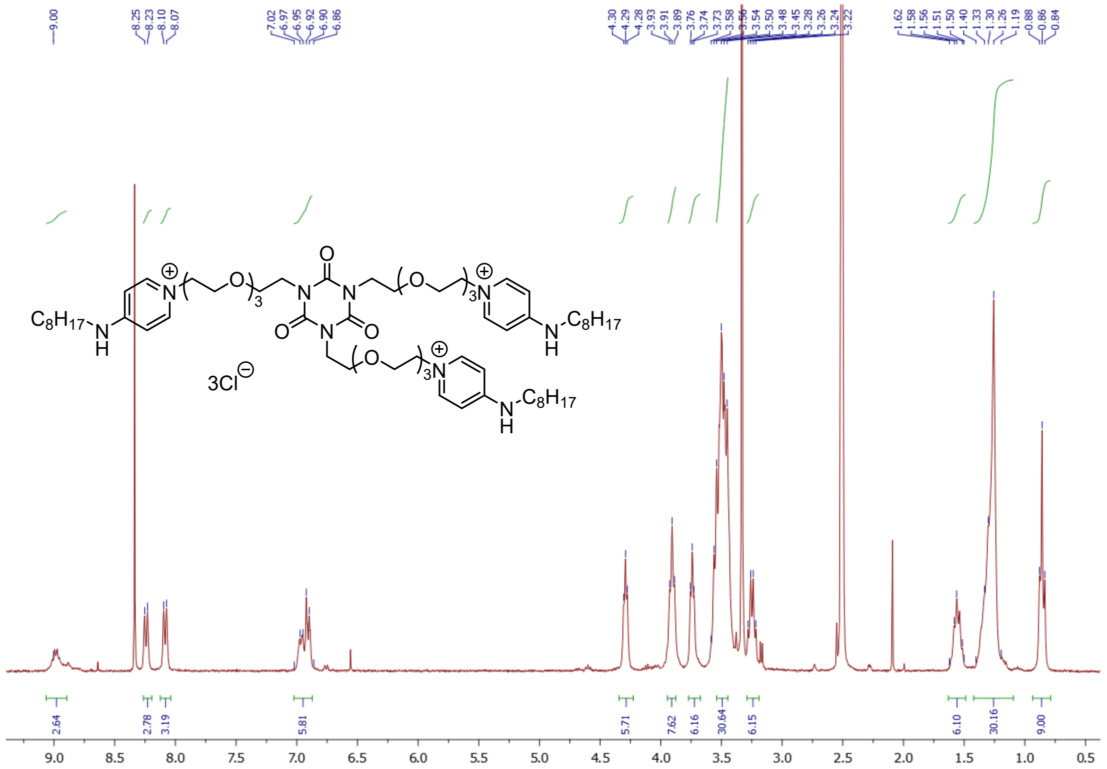

^{13}C NMR spectrum of **9a**

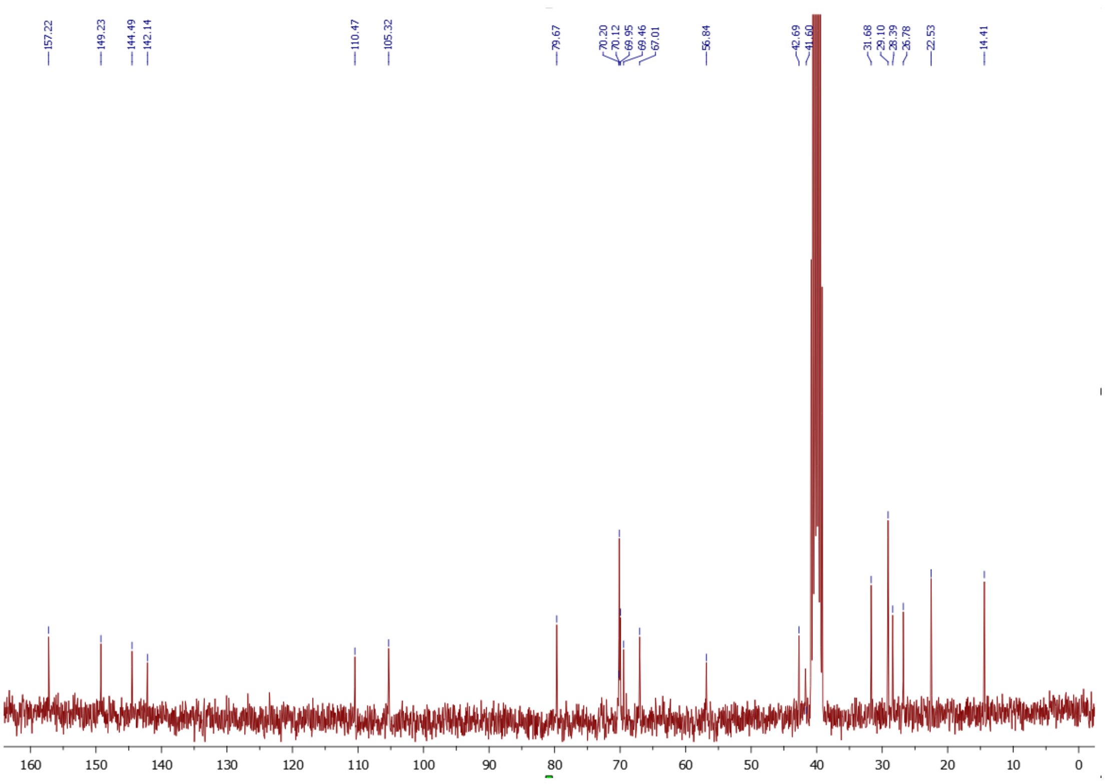

HRMS of **9a**

Display Report

Analysis Info

Analysis Name	D:\Data\Kolotyrkina\2023\Seferyan\0601028.d		
Method	tune_wide.m	Operator	BDAL@DE
Sample Name	/VERS MC-047	Instrument / Ser#	micrOTOF 10248
Comment	C66H114N9O12 mH1225.8659 calibrant added CH3OH		

Acquisition Parameter

Source Type	ESI	Ion Polarity	Positive	Set Nebulizer	0.4 Bar
Focus	Not active			Set Dry Heater	180 °C
Scan Begin	50 m/z	Set Capillary	4500 V	Set Dry Gas	4.0 l/min
Scan End	3000 m/z	Set End Plate Offset	-500 V	Set Divert Valve	Waste

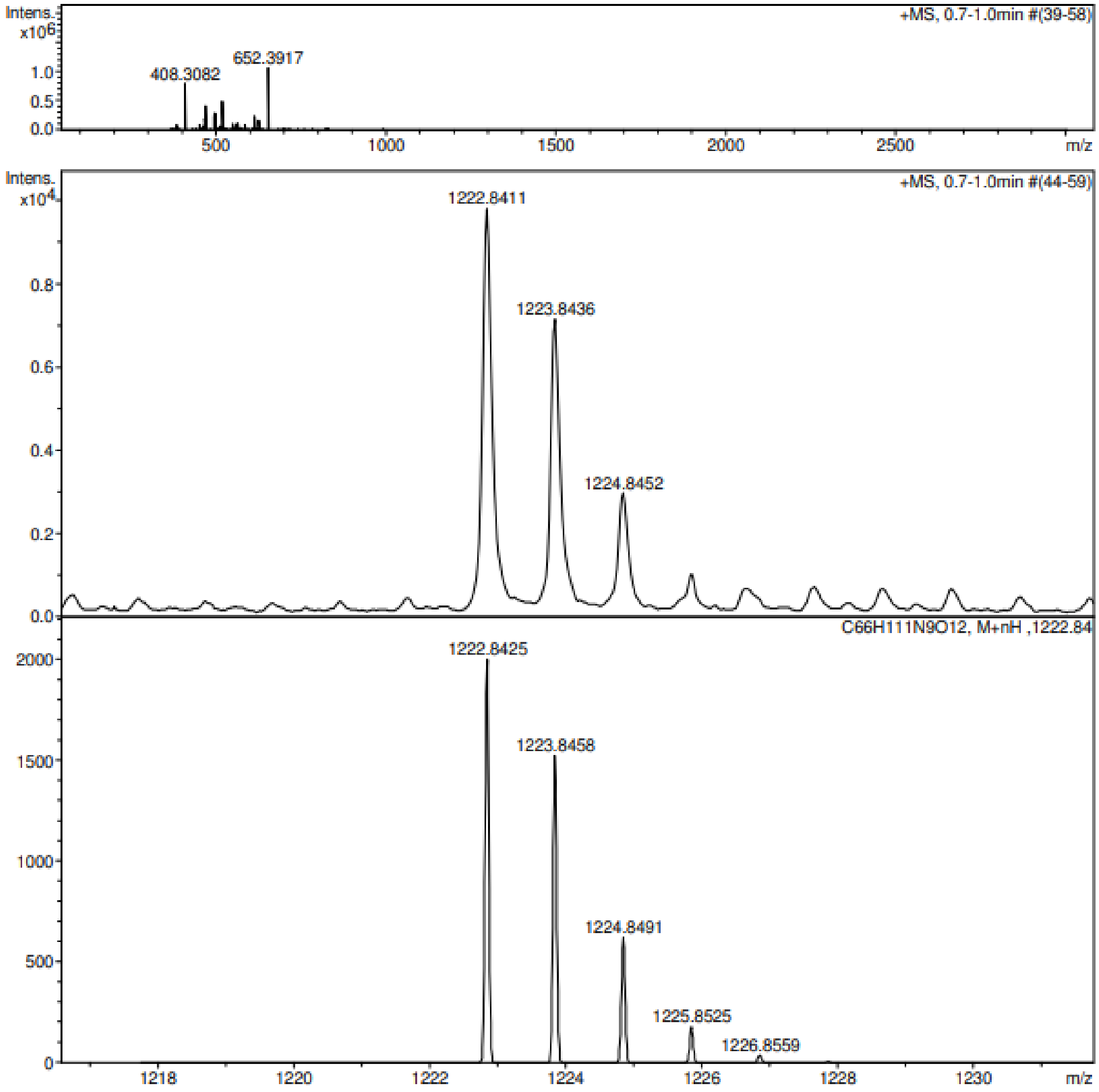

^{1}H NMR spectrum of 1,1',1''-((((((((2,4,6-trioxo-1,3,5-triazinane-1,3,5-triyl)tris(ethane-2,1-diyl))tris(oxy))tris(ethane-2,1-diyl))tris(oxy))tris(ethane-2,1-diyl))tris(oxy))tris(ethane-2,1-diyl))tris(4-(decylamino)pyridin-1-ium) **(9b)**

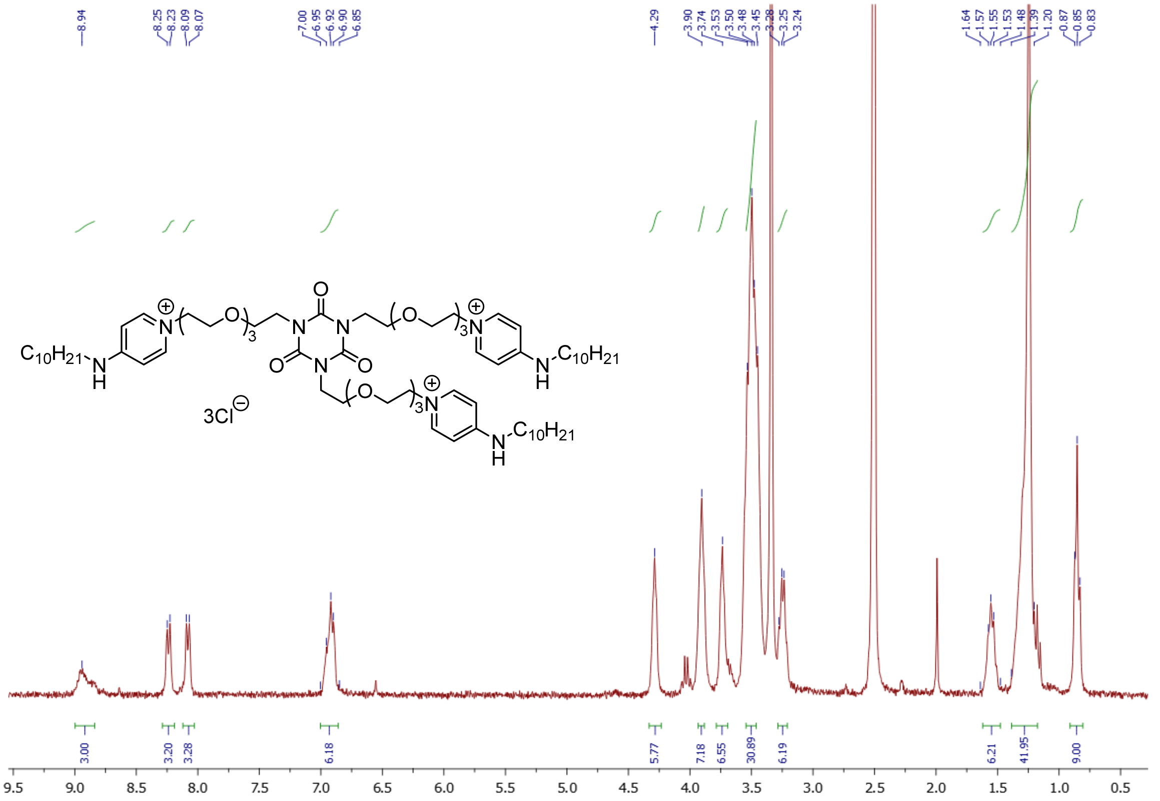

^{13}C NMR spectrum of **9b**

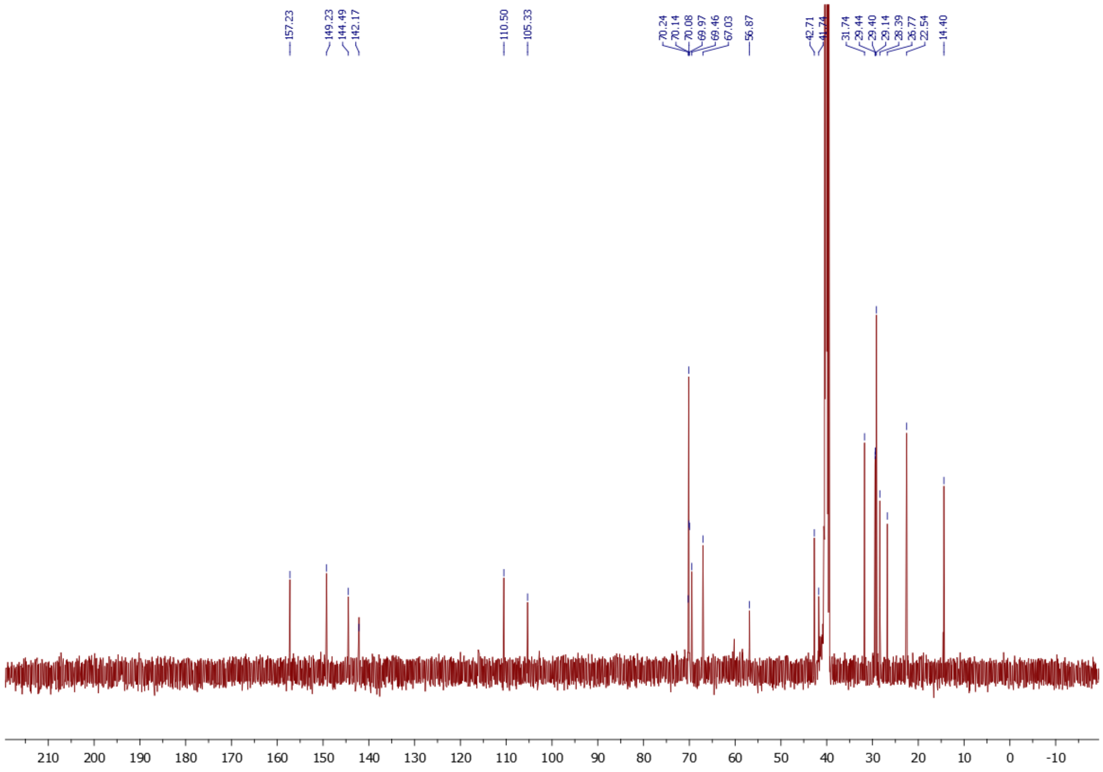

HRMS of **9b**

Display Report

Analysis Info

Analysis Name	D:\Data\Kolotyrkina\2023\Seferyan\0601029.d		
Method	tune_wide.m	Operator	BDAL@DE
Sample Name	/VERS MC-046	Instrument / Ser#	micrOTOF 10248
Comment	C72H126N9O12 mH1309.9598 calibrant added CH3OH		

Acquisition Parameter

Source Type	ESI	Ion Polarity	Positive	Set Nebulizer	0.4 Bar
Focus	Not active			Set Dry Heater	180 °C
Scan Begin	50 m/z	Set Capillary	4500 V	Set Dry Gas	4.0 l/min
Scan End	3000 m/z	Set End Plate Offset	-500 V	Set Divert Valve	Waste

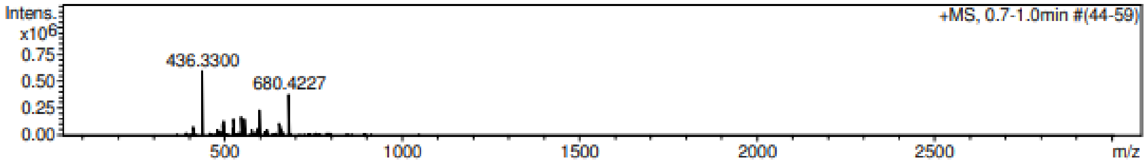

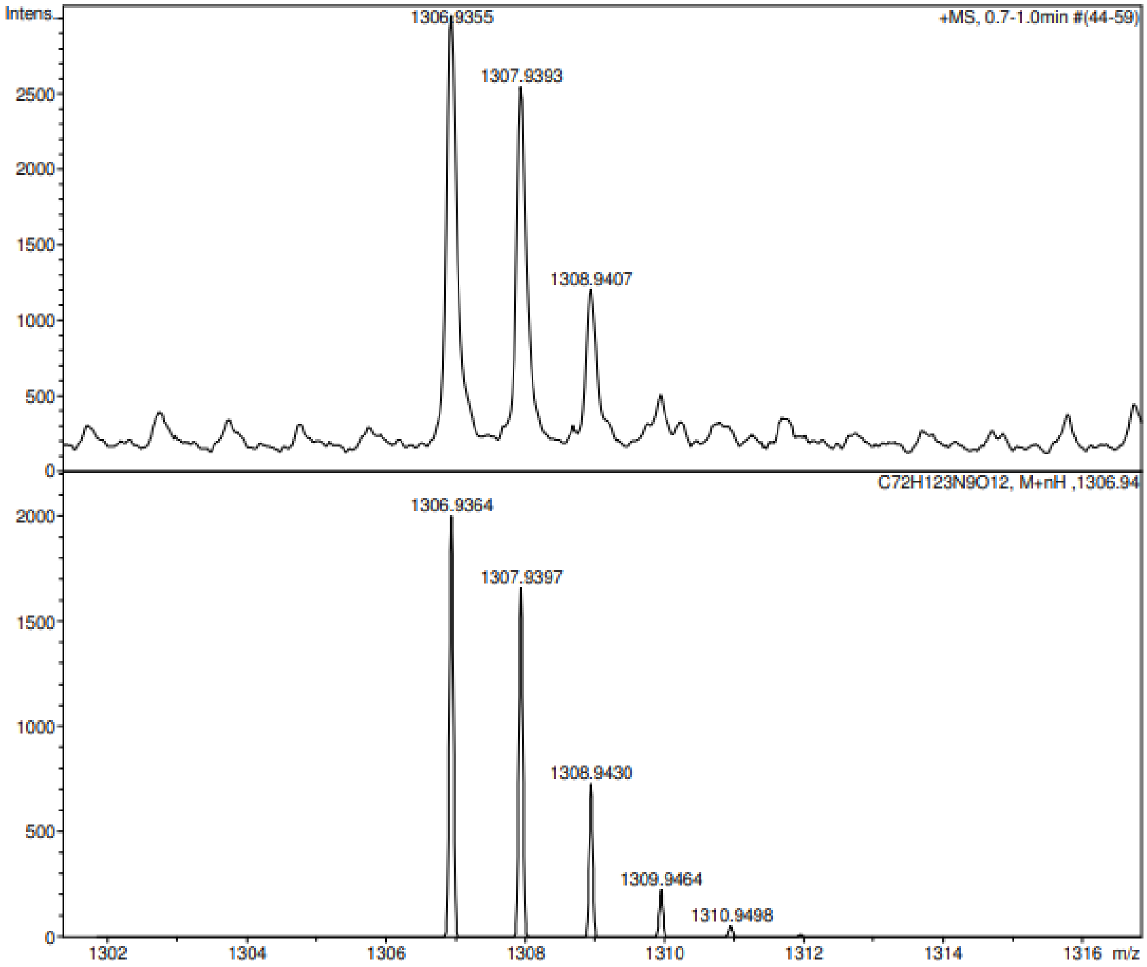

[1]**H NMR** spectrum of 1,1',1''-((((((((2,4,6-trioxo-1,3,5-triazinane-1,3,5-triyl)tris(ethane-2,1-diyl))tris(oxy))tris(ethane-2,1-diyl))tris(oxy))tris(ethane-2,1-diyl))tris(oxy))tris(ethane-2,1-diyl))tris(4-(dodecylamino)pyridin-1-ium) **(9c)**

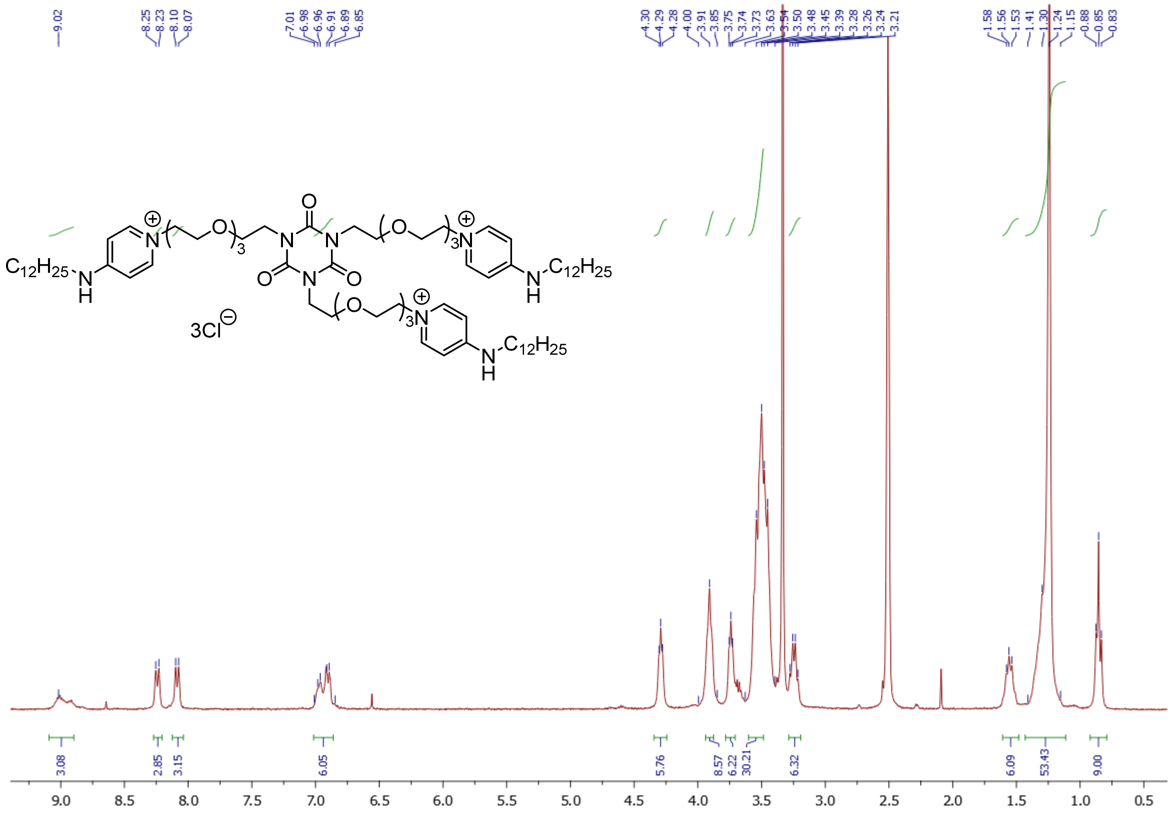

[13]**C NMR** spectrum of **9c**

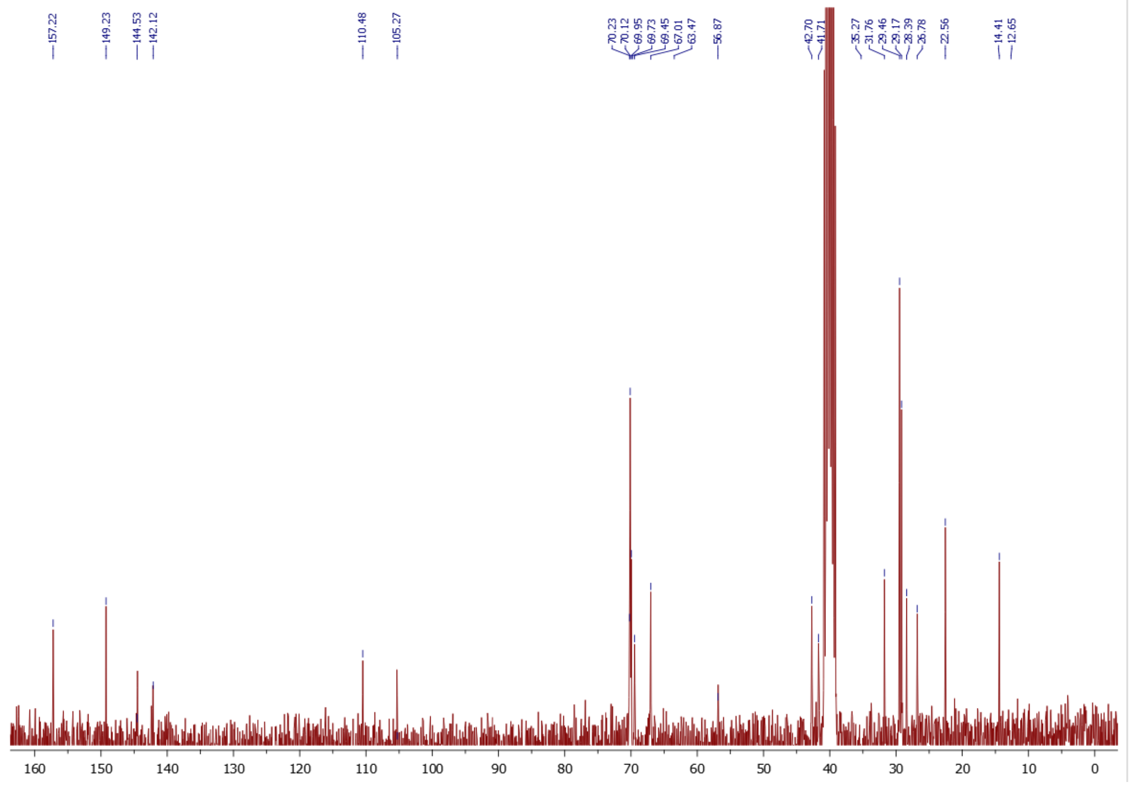

HRMS of 9c

Display Report

Analysis Info

Analysis Name	D:\Data\Kolotyrkina\2023\Seferyan\0601027.d		
Method	tune_wide.m	Operator	BDAL@DE
Sample Name	/VERS MC-048	Instrument / Ser#	micrOTOF 10248
Comment	C78H138N9O12 mH1394.0537 calibrant added CH3OH		

Acquisition Parameter

Source Type	ESI	Ion Polarity	Positive	Set Nebulizer	0.4 Bar
Focus	Not active			Set Dry Heater	180 °C
Scan Begin	50 m/z	Set Capillary	4500 V	Set Dry Gas	4.0 l/min
Scan End	3000 m/z	Set End Plate Offset	-500 V	Set Divert Valve	Waste

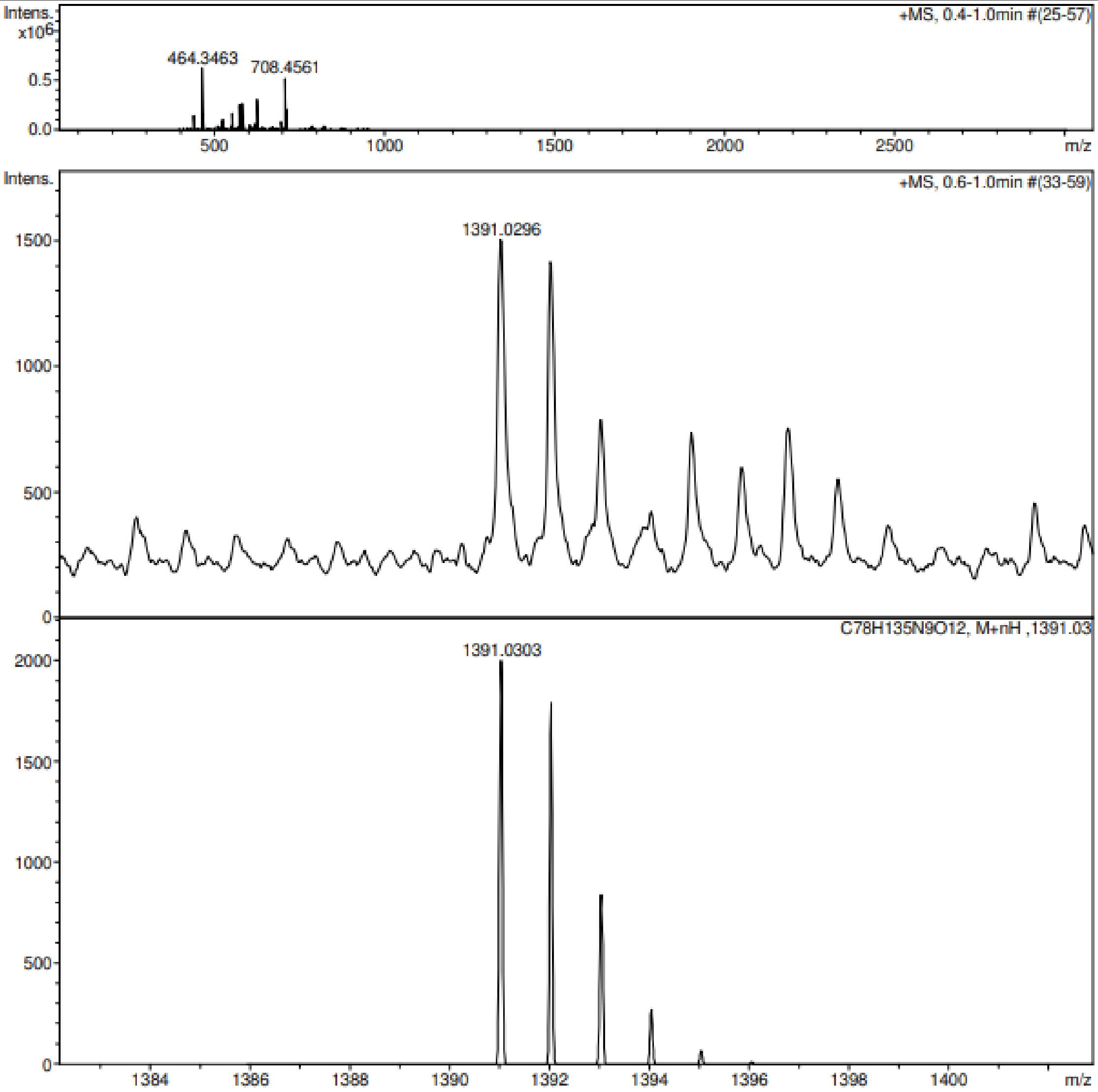

2D NMR spectra of the compound 7g

Figure S1. The structure and numbering of the compound **7g**. HMBC is shown with red curves; COSY is shown with blue curves.

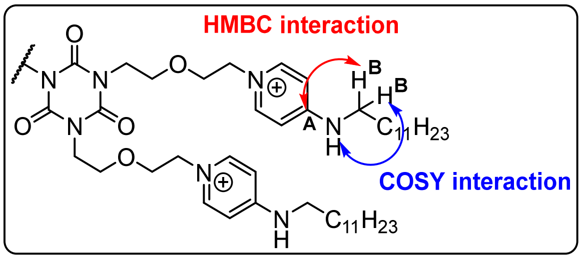

Structure A (confirmed)

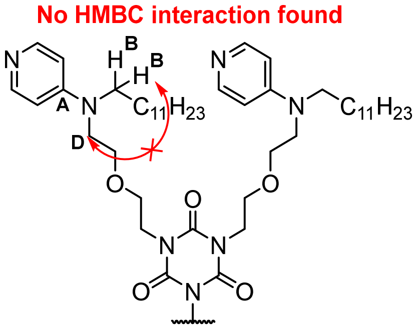

Structure B

The structure of the compound **7g** was confirmed using 1D NMR (page S19) and 2D NMR spectroscopy (pages S33-S34). The assignment was performed by means of 2D ^{1}H-^{13}C HSQC, ^{1}H-^{13}C HMBC and ^{1}H-^{1}H COSY spectra. The key interactions for alkylation route confirmation (**Structure A** vs **Structure B**) are described below.

2D ^{1}H-^{1}H COSY spectra gave overlapped signals between NH-group (9.05-9.20 ppm) and CH_2^{B}-group (3.21 ppm). The cross-peak found suggests the presence of secondary amine as in **Structure A**. 2D ^{1}H-^{13}C HMBC spectra showed heteronuclear correlation among aromatic CH_2^{B}-group (3.21 ppm) and C^{A} (157.1 ppm). The signal was seen in reflection since the ^{13}C NMR region ended at 150 ppm. However, no cross-peaks were visualized between CH_2^{B}-group (3.21 ppm) and C^{D} (56.9 ppm) as it would be in the **Structure B**. Thus, the formation of **Structure A** during alkylation is confirmed by NMR analysis.

^{1}H-^{13}C HMBC NMR spectrum of the compound 7g

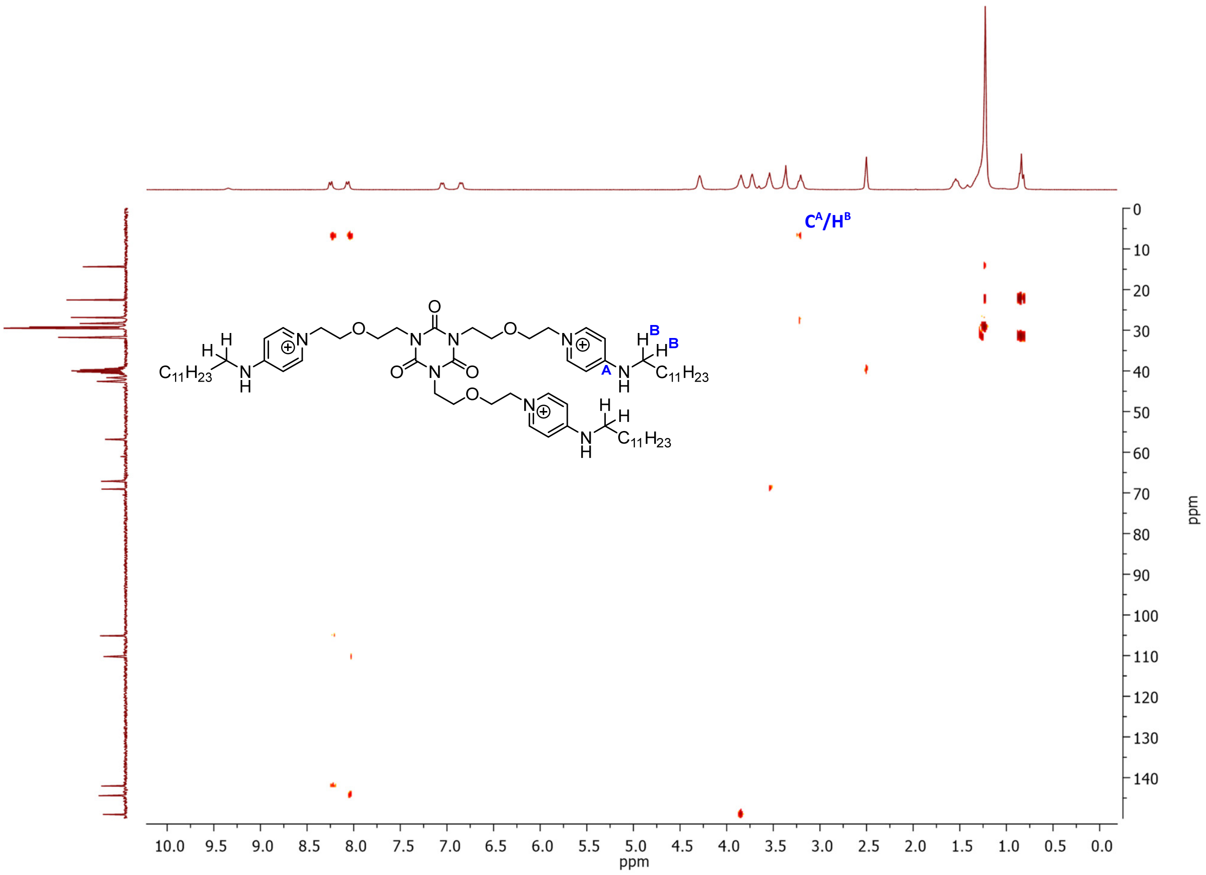

^{1}H-^{1}H COSY NMR spectrum of the compound 7g

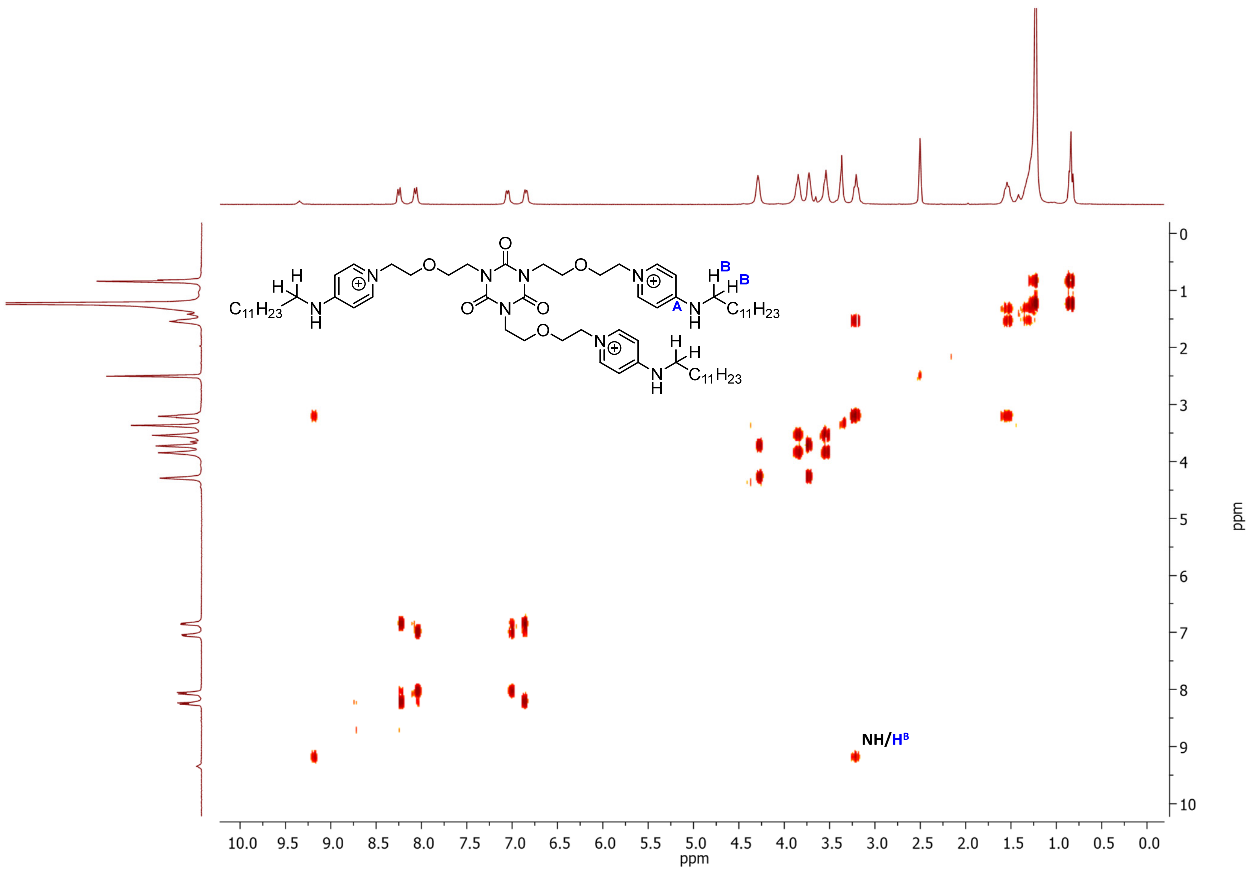

^{1}H-^{13}C HSQC NMR spectrum of the compound 7g

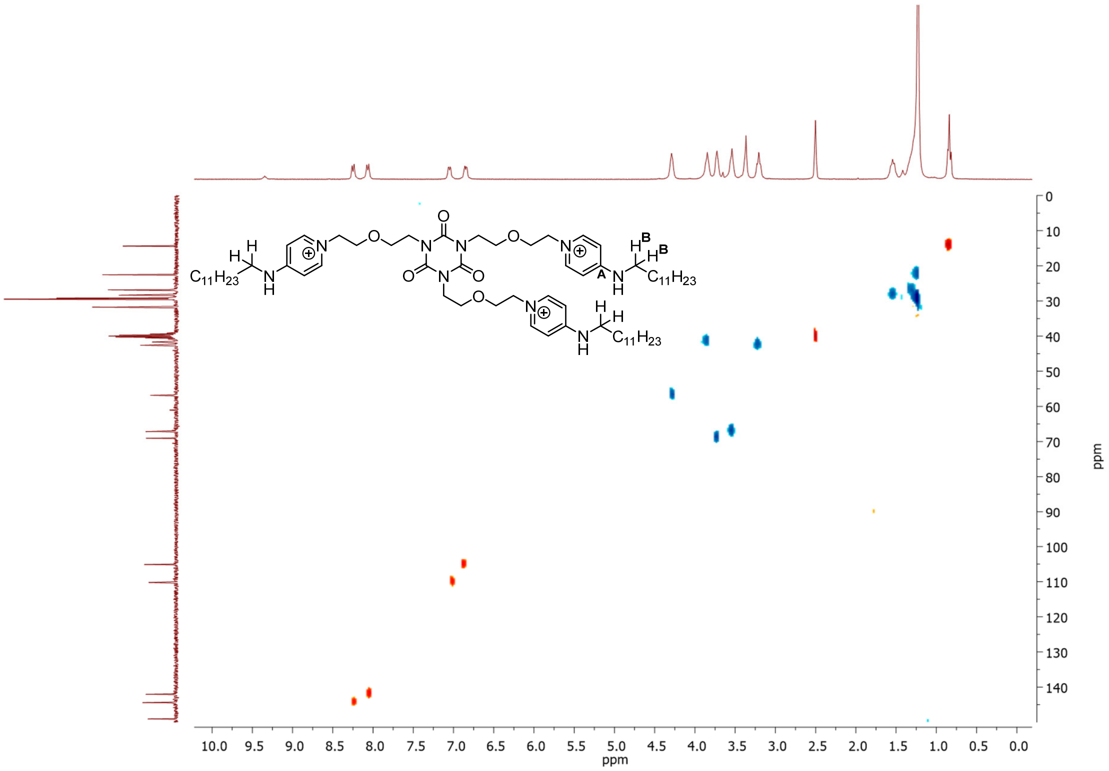

Figures and tables

Figure S2. General formula of the newly synthesized trimeric QACs.

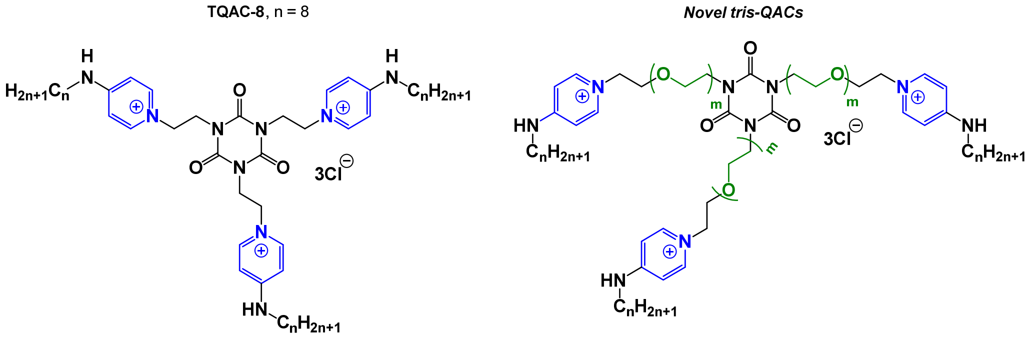

Table S1. Solubility in Water of novel tris-QACs

Compounds	Solubility in water (mg/mL)
7c: m=1, n=7	250
7e: m=1, n=9	195
7g: m=1, n=11	105
8a: m=2, n=7	300
8b: m=2, n=9	250
8c: m=3, n=11	195
9b: m=3, n=9	370
TQAC-8	55

Figure S3. General formula of the isomers synthesized trimeric QACs.

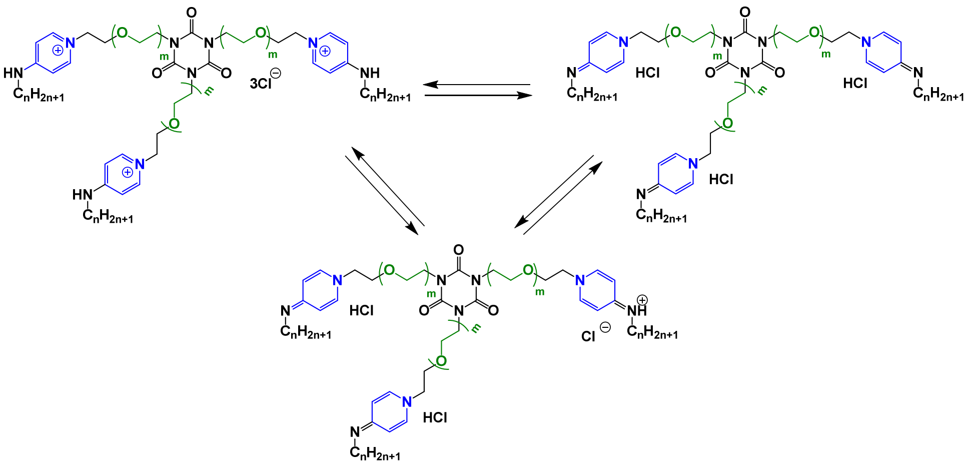

Figure S4. Example of MICs evaluation: horizontal row – two-fold dilutions (500-0.25 mg/L) of the biocides in nutrient broth; vertical row – series of tested biocides; MIC is highlighted in a blue circle; samples for MBC evaluation is highlighted in red.

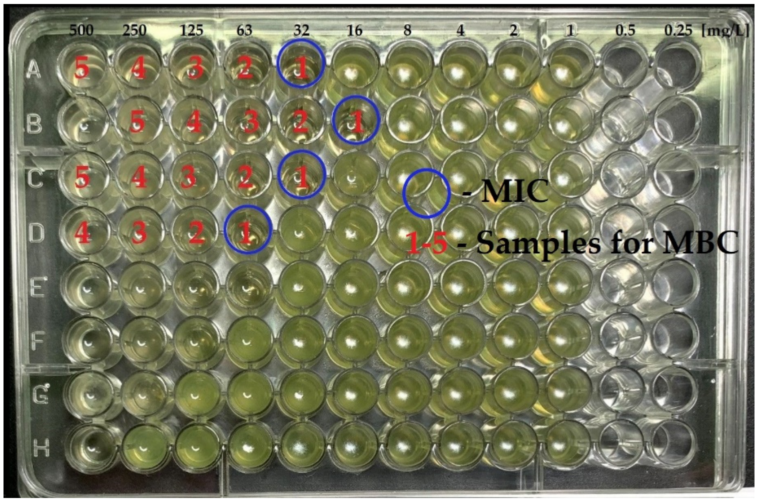

Figure S5. Example of MBCs evaluation: horizontal row – seeding of test samples from MIC evaluation (Figure S1) on a nutrient medium for bacterial growth; vertical row – series of tested biocides; MBC is highlighted in a blue circle.

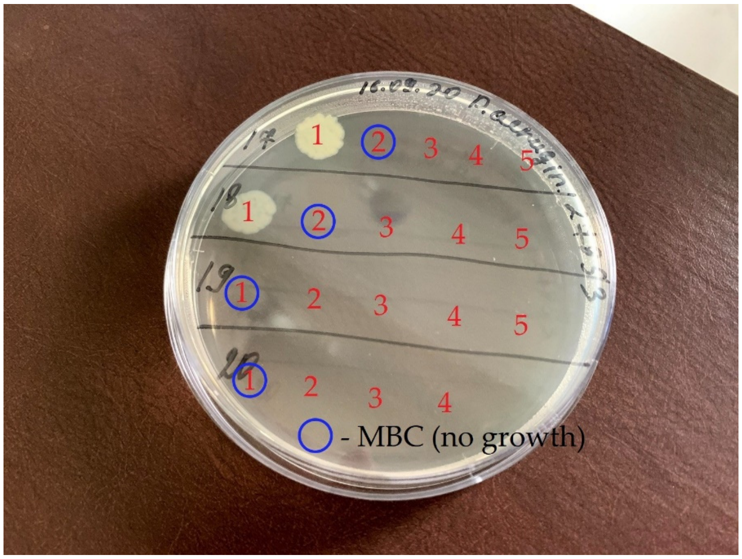

Figure S6**.** Examples of MICs evaluation for compound **7d**: top left – *S. aureus* B-8648 (MIC = 2 mg/L); top right – *E. coli* B-3421/19 (MIC = 16 mg/L); bottom left – *K. pneumoniae* B-2523/18 (MIC = 63 mg/L); bottom right – *P. aeruginosa* B-2099/18 (MIC = 8 mg/L); MIC is highlighted in a blue circle; two-fold dilutions (500-1 mg/L) of the novel biocide **7d** is highlighted in red.

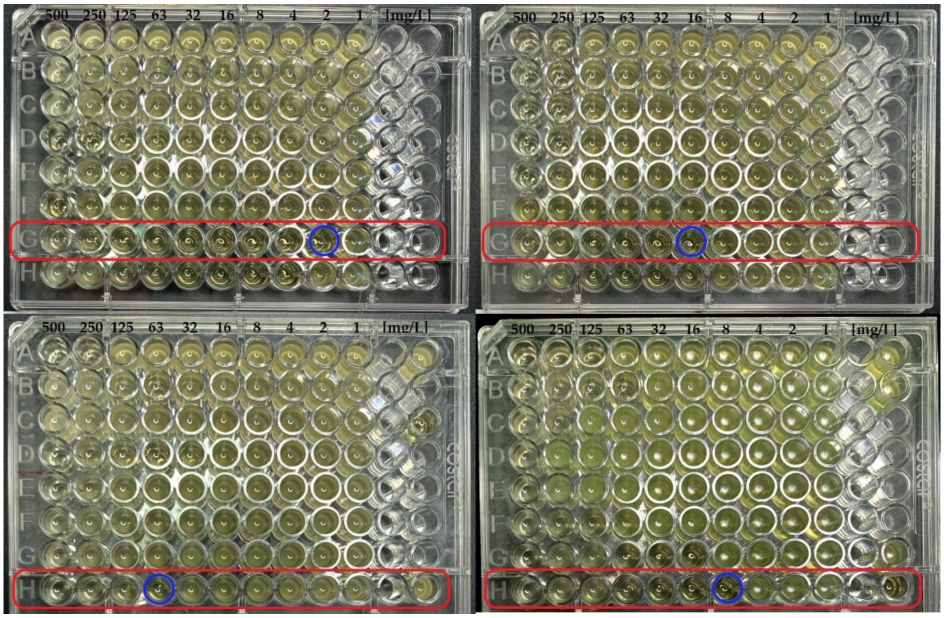

Supplement: Supplementary file 1 [file ijms-24-10512-s001.zip › ijms-2354647-supplementary.pdf]
